# Interpersonal brain synchronization during face-to-face economic exchange between acquainted dyads

**DOI:** 10.1093/oons/kvad007

**Published:** 2023-06-21

**Authors:** Yuto Kikuchi, Kensuke Tanioka, Tomoyuki Hiroyasu, Satoru Hiwa

**Affiliations:** Graduate School of Life and Medical Sciences, Doshisha University, 1-3 Tatara Miyakodani, Kyotanabe, Kyoto610-0394, Japan; Department of Biomedical Sciences and Informatics, Doshisha University, 1-3 Tatara Miyakodani, Kyotanabe, Kyoto 610-0394, Japan; Department of Biomedical Sciences and Informatics, Doshisha University, 1-3 Tatara Miyakodani, Kyotanabe, Kyoto 610-0394, Japan; Department of Biomedical Sciences and Informatics, Doshisha University, 1-3 Tatara Miyakodani, Kyotanabe, Kyoto 610-0394, Japan

**Keywords:** interpersonal brain synchronization, face-to-face economic exchange, hyperscanning, acquaintanceship, fNIRS, human sociality

## Abstract

Interpersonal brain synchronization (IBS) has been observed during social interactions and involves various factors, such as familiarity with the partner and type of social activity. A previous study has shown that face-to-face (FF) interactions in pairs of strangers increase IBS. However, it is unclear whether this can be observed when the nature of the interacting partners is different. Herein, we aimed to extend these findings to pairs of acquaintances. Neural activity in the frontal and temporal regions was recorded using functional near-infrared spectroscopy hyperscanning. Participants played an ultimatum game that required virtual economic exchange in two experimental settings: face-to-face and face-blocked conditions. Random pair analysis confirmed whether IBS was induced by social interaction. Contrary to the aforementioned study, our results did not show any cooperative behavior or task-induced IBS increase. Conversely, the random pair analysis results revealed that the pair-specific IBS was significant only in the task condition at the left and right superior frontal, middle frontal, orbital superior frontal, right superior temporal, precentral and postcentral gyri. Our results tentatively suggested that FF interaction in acquainted pairs did not increase IBS and supported the idea that IBS is affected by ‘with whom we interact and how’.

## INTRODUCTION

Face-to-face (FF) communication plays a vital role in building trust and cooperation with others. Cooperative behavior is also found in other animals, but large-scale and stable cooperative behavior is unique to humans [1]. What makes such a cooperation possible is the unique human ability to empathize with others and to infer their feelings and intentions [2]. It is essential to investigate how our brain functions are associated with social interaction to better understand human sociality.

In the recent years, hyperscanning, a method of simultaneously measuring the brain activity of two or more people, has been used in many studies to investigate the neural basis of social interaction [3]. It has been successfully used in various neuroimaging modalities, such as functional magnetic resonance imaging (fMRI), functional near-infrared spectroscopy (fNIRS) and electroencephalography [4–8, 41] . Many researchers have used these modalities to investigate various social behaviors, such as cooperation, competition, empathy and trust. Various experimental paradigms have been proposed to experimentally reproduce such social behavior, including coordination tasks in which participants synchronize their movements [9, 10], eye contact and joint attention tasks [11, 12], economic game tasks, such as the prisoner's dilemma and ultimatum games [13, 14] and tasks involving everyday activities, such as choral singing and discussions [7, 15]. Even though the experimental paradigm is different, previous studies have confirmed that interpersonal brain synchronization (IBS) in the dorsolateral prefrontal cortex and the temporoparietal junction (TPJ) is the key feature of social behavior.

Gvirts and Perlmutter [16] proposed that IBS during social interaction can be affected by three factors: (1) the type of social activity, e.g. IBS is more likely to occur in interactional social activities than in non-interactional social activities. Also, the level of interaction required mediates the level of IBS; (2) IBS increases in the setting of the interaction, e.g. when facing a partner; and (3) IBS appears stronger in the nature of the interaction partner, e.g. between male and female lovers, than in male and female friends or strangers. Among these factors, Tang et al. [17] focused on the setting of interactions, investigating its effect on IBS using an ultimatum game under two different environmental settings: the FF and face-blocked (FB) conditions. They found that FF interactions increased ‘shared intentionality’, a positive belief in each other’s cooperative decision making, compared with FB interactions, and facilitated cooperation between partners. In addition, fNIRS-based hyperscanning demonstrated that IBS in the right TPJ was greater in FF interactions than in FB interactions and was also increased by the existence of shared intentionality between partners. Their work provides important insights, as they observed IBS between two individuals during FF interactions. However, it should be noted that these findings were confirmed for pairs of strangers, and it remains unclear whether these results can be reproduced when the nature of the interacting partners is different. It is also of great interest whether the nature of the interacting partners affects the increased IBS in FF interactions.

Herein, we investigated how FF interaction affects IBS in pairs of acquaintances. We used the same experimental paradigm as Tang et al. [17], intending to extend their findings in stranger pairs to acquainted interacting partners. We investigated the following hypotheses: (1) FF interaction may potentially enhance shared intentionality between pairs and promote cooperation, as it offers more visual information about the partner compared to FB interaction; (2) IBS in the right TPJ, responsible for inferring the partner's intentions, is greater in FF interaction than in FB interaction. These hypotheses are the same as in the original study, and we aimed to replicate their findings in acquainted pairs. In addition to the original study, we examined whether IBS was specific to the interacting pair. One of the pitfalls of hyperscanning is that an increased IBS might be observed when a different participant performs the same task independently, although there is no interaction between pairs [18]. Therefore, we confirmed that IBS would be higher in interacting pairs than in non-interacting pairs by performing random-pair analysis.

## MATERIALS AND METHODS

### Participants

Forty-eight healthy university students (24 same-sex pairs; FF: 12 pairs with 10 pairs of men, age [mean ± standard deviation]: 22.00 ± 1.12; FB: 12 pairs with 10 pairs of men, age: 22.46 ± 1.41) participated in our study. All pairs belonged to the same laboratory at the time of the study and were acquainted before the experiment; they recognized each other's names and faces and had daily conversations. It should be noted that we could not control for the sample size and sampling bias, such as social distance. All participants were informed of the methods and risks of the experiment and provided written informed consent.

### Procedure and behavioral data acquisition

**Figure 1:**
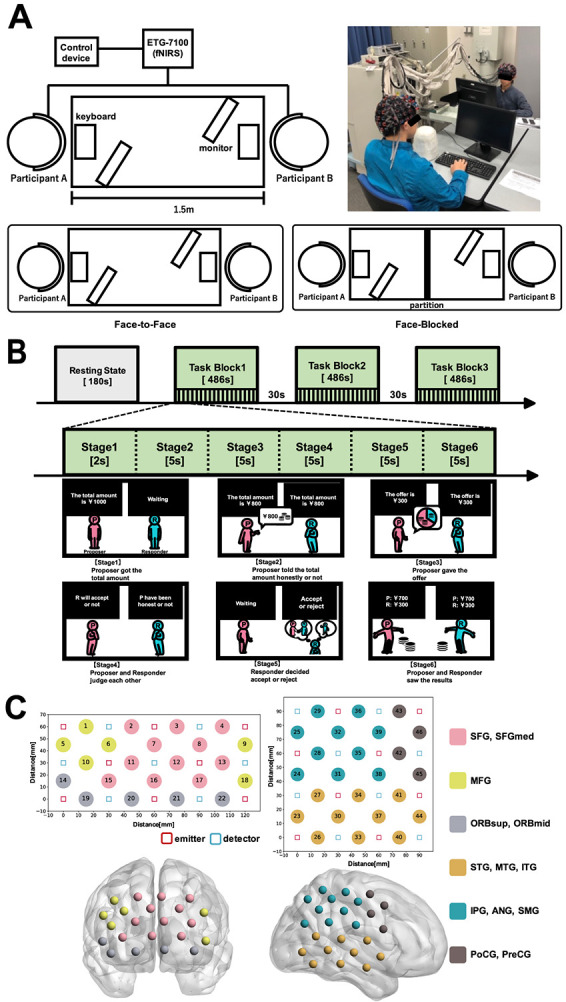
**Experimental design and setup.** (A) Experimental environment. FF condition: The pairs were opposite each other and could see each other’s faces. FB condition: The pairs were opposite each other but separated by a partition wall. (B) Timeline of the experimental protocol. Resting-state block: participants were instructed to be relaxed with their eyes closed and remain as still as possible. Task block: one task block consisted of 18 consecutive trials. In one trial, the pairs played one ultimatum game. There were three task blocks, and consequently, the pairs performed 54 rounds of the ultimatum game in total. (C) fNIRS probe assignment. The emitters and detectors are indicated as squares, respectively. The measurement channels were marked as circles and numbered. Anatomical regions were determined by the virtual registration method. See Table 1 for spatial registration of the fNIRS channel location to the automated anatomical labeling atlas [42] in MNI space. Abbreviations: FF, face-to-face; FB, face-blocked; fNIRS, functional near-infrared spectroscopy; MNI, Montreal neurological institute

Each participant sat at a table with a keyboard and a display. Each pair was randomly assigned to one of two experimental environments: FF or FB. In the FF condition, the pair could see each other's faces (Fig. 1A). The proposer communicated only information about the reward amount and the offer verbally. The responder was instructed not to say anything at all. In the FB condition, the pairs were separated by a partition wall and could not see each other's faces. Under both conditions, the decisions in the task were performed by pressing the keyboard. Information related to the task, such as the reward amount and questions, was displayed on the screen.

The task was an ultimatum game with the same design as that used by Tang et al. [17]. The difference from the original study was the use of the Japanese monetary unit in representing the reward. Before starting the experiment, each participant was randomly assigned to either the role of a proposer or a responder. This role was maintained until the end of the experiment. The original study added the following two stages to the traditional ultimatum game [19]: (1) a stage where the proposer tells the responder the amount of reward (the proposer can deceive the responder by stating a false amount), and (2) a stage where the proposer judges whether the responder accepts his offer, and the responder judges whether the proposer has reported honestly. Adding these steps allowed us to measure how well the pairs could understand the other's intentions.

Each pair played 54 rounds of the ultimatum game together, divided into three task blocks, each block lasting approximately 8 minutes. One ultimatum game consisted of six stages (Fig. 1B). In Stage 1, the Proposer received one of six different rewards (¥100, \200, \500, \1000, \1500 and \2000). In Stage 2, the proposer tells the responder the reward received. At this time, the proposer was able to deceive the respondent by giving a false amount (honesty rate [HR]: the percentage of the reward reported to the responder compared with the amount received by the proposer). In Stage 3, the proposer decides how much of the reward is distributed to the responder. The percentage of the amount distributed to the responder (the offer) to the amount received by the proposer (the actual total amount) was defined as the offer proportion (OP). In addition, the percentage of trials in which exactly 50% of the reward amount told by the proposer to the responder was distributed was defined as the fairness rate (FR). It should be noted that in Stages 2 and 3, the proposer was also instructed to verbally communicate (in the FF condition only) the total amount (in stage 2) and the transfer amount (in stage 3) besides pressing the button as the response. In stage 4, the participants evaluated each other's actions. The proposer judged whether the responder would accept the offer or not, and the responder judged whether the proposer had honestly reported the amount received. The percentage of trials in which the pair judged each other's behavior positively (i.e. the Proposer judged that the Responder would accept the offer and the responder judged that the proposer told the truth) was defined as the shared intentionality rate (SIR). In Stage 5, the responder decided whether to accept the offer. The percentage of trials in which the responder rejected the offer was defined as the rejection rate (RR). If the responder accepted the offer, both players received the reward as distributed by the Proposer. Otherwise, no one received rewards. In stage 6, the true winnings of each participant were shown (for example, the proposer received ¥1000, told the Responder that the reward amount was ¥800, and distributed ¥400. In this case, the display will show ‘proposer: ¥600, responder: ¥400’). To measure emotional effects during the interaction, participants were asked to complete the Positive and Negative Affect Schedule (PANAS) scale after the experiment. It is important to note that the game gain, the total amount of rewards, was calculated as a percentage (%) of the maximum possible reward of ¥47 700.

During the experimental setup, we ensured that participants' facial expressions were visible. We instructed those in the FF condition to using the partner's expression as a clue to answer the questions in stage 4. However, we instructed participants to minimize any bodily movements except for pressing the keyboard to avoid the influence of the motion artifact on the results, except for physiological factors.

### fNIRS data acquisition

An ETG-7100 optical topography system (Hitachi, Ltd., Tokyo, Japan) was used to record the concentration changes in oxyhemoglobin (HbO) and deoxyhemoglobin (HbR) for each pair (Fig. 1 C). The absorption of near-infrared light (wavelength: 695 ± 20 nm and 830 ± 20 nm, light intensity: 2 mW) was measured at a sampling rate of 10 Hz. Two probe sets were fitted to each subject: a 3 × 5 optode probe set (eight emitters and seven detectors, optode distance: 30 mm, 22 channels in total) and a 4 × 4 optode probe set (eight emitters and eight detectors, optode distance: 30 mm, 24 channels in total). The 3 × 5 probe set was attached to the frontal region, and the 4 × 4 probe set was attached to the right temporoparietal region according to the reference points of the International 10–20 System. Also, we confirmed the sensor sensitivity with the calibration function of the NIRS device before measuring and excluded the effect of bad channels.

The measured data were converted into HbO and HbR concentration changes using the modified Beer–Lambert's law. A three-dimensional digitizer (Patriot™, Polhemus) was used to record the exact spatial coordinates of five reference points (nasion, ion, top, left ear, right ear) and 31 optical probes. To investigate the correspondence between the fNIRS channels and the brain regions, the anatomical regions [20] corresponding to each channel were estimated using the virtual registration method [21] as shown in Table 1. It should be noted that we could use the fNIRS device that offered more channels than the one used in the original study. Performing measurements with a greater number of channels and an extended range can be very effective in reducing the influence of individual differences in head and brain region size and avoiding false negative detections. Therefore, we chose to conduct measurements over a wider range. Additionally, we confirmed from the virtual registration results that our probe placement covers all brain regions measured in the original study.

**Table 1:** Measurement channels, group-averaged montreal neurological institute (MNI) coordinates (n = 48), anatomical regions, and region probabilities

Channel	MNI coordinates			Anatomical region	Probability
	x	y	z		
1	40.667	40.667	40.667	MFG.R	1
2	19	53	44	SFG.R	0.809
				SFGmed.R	0.191
3	−2.667	54.333	44.333	SFGmed.L	0.752
				SFGmed.R	0.142
				SFG.L	0.106
4	−28.667	45.333	42.667	SFG.L	0.539
				MFG.L	0.461
5	50.667	40	27	MFG.R	0.675
				IFGtriang.R	0.325
6	31.667	56	31	MFG.R	0.826
				SFG.R	0.174
7	7.333	63.667	33.333	SFGmed.R	0.613
				SFGmed.L	0.248
				SFG.R	0.139
8	−19	60.333	33.333	SFG.L	0.8
				MFG.L	0.1
				SFGmed.L	0.1
9	−41.667	46	29.667	MFG.L	1
10	43.333	56.333	17.667	MFG.R	0.971
11	21.333	68.667	21.333	SFG.R	0.74
				SFGmed.R	0.132
				MFG.R	0.128
12	−5	68.667	21.667	SFGmed.L	0.695
				SFG.L	0.255
13	−30.333	62.333	20	SFG.L	0.534
				MFG.L	0.466
14	51.333	49	3.333	MFG.R	0.446
				IFGtriang.R	0.317
				ORBinf.R	0.137
				ORBmid.R	0.101
15	33	66.667	7	SFG.R	0.661
				MFG.R	0.273
16	6	72.333	8.333	SFGmed.R	0.727
				SFGmed.L	0.227
17	−21	71	8	SFG.L	0.977
18	−43.333	56.667	3.667	MFG.L	0.634
				ORBmid.L	0.303
19	43.667	59.667	−7.667	ORBmid.R	0.929
20	21	71.333	−6	ORBsup.R	0.536
				SFG.R	0.212
				ORBmid.R	0.17
21	−5.333	71.667	−4.333	ORBmid.L	0.586
				SFGmed.L	0.217
				ORBsup.L	0.134
22	−32.667	65.667	−5.333	ORBmid.L	0.548
				SFG.L	0.265
				ORBsup.L	0.163
23	58.333	−70.667	−1	MTG.R	0.444
				ITG.R	0.352
				MOG.R	0.137
24	57.667	−68.333	26.667	ANG.R	0.429
				MTG.R	0.353
				MOG.R	0.218
25	50.333	−65.333	50.333	ANG.R	0.861
				IPL.R	0.139
26	63.333	−60		ITG.R	0.942
27	65.333	−57.333	14.333	MTG.R	0.794
				STG.R	0.189

(Continued)

**Table 1:** Continued

Channel	MNI coordinates			Anatomical region	Probability
	x	y	z		
28	61.667	−54.333	40.333	IPL.R	0.468
				ANG.R	0.415
				SMG.R	0.117
29	48.333	−50.667	59.333	IPL.R	0.574
				SPG.R	0.41
30	71	−45.667	1	MTG.R	0.971
31	68	−44.667	28	SMG.R	0.545
				STG.R	0.318
				ANG.R	0.137
32	61.667	−42	50.667	IPL.R	0.68
				SMG.R	0.32
33	72	−33.333	−12.333	MTG.R	0.681
				ITG.R	0.319
34	72	−32.667	11.667	STG.R	0.82
				MTG.R	0.18
35	69	−30	39	SMG.R	1
36	55.667	−28.333	57.333	PoCG.R	0.451
				IPG.R	0.292
				SMG.R	0.156
				SPG.R	0.101
37	73	−20.333	−1.333	STG.R	0.588
				MTG.R	0.413
38	70	−18.333	25.333	SMG.R	0.663
				PoCG.R	0.254
39	62.667	−16.333	48	PoCG.R	0.625
				SMG.R	0.313
40	70	−8	−16	MTG.R	0.87
				STG.R	0.13
41	69	−7.333	10.333	STG.R	0.547
				PoCG.R	0.264
				ROL.R	0.173
42	65.667	−3.667	36.333	PoCG.R	0.831
				PreCG.R	0.169
43	53.667	−4.667	55.333	PreCG.R	0.492
				MFG.R	0.418
44	64.333	5.667	−5.333	TPOsup.R	0.551
				STG.R	0.377
45	66	8.333	21.333	PreCG.R	0.485
				PoCG.R	0.21
				IFGoperc.R	0.164
				ROL.R	0.131
46	56.333	9.333	44.333	PreCG.R	0.767
				MFG.R	0.178

Abbreviations: MFG, middle frontal gyrus; SFG, superior frontal gyrus; SFGmed, superior frontal gyrus, medial; IFGoperc, inferior frontal gyrus opercular part; IFGtriang, inferior frontal gyrus triangular part; ORBmid, middle frontal gyrus, orbital; ORBsup, superior frontal gyrus, orbital; STG, superior temporal gyrus, MTG, middle temporal gyrus; ITG, inferior temporal gyrus; TPOsup, temporal pole: superior temporal gyrus; ANG, angular gyrus; IPG, inferior parietal gyrus; SMG, supramarginal gyrus; PreCG, precentral gyrus; PoCG, postcentral gyrus; R, right; L, left; ROL, Rolandic operculum.

### Data analysis

#### Behavioral data

The six variables, HR, OP, FR, SIR, RR, game gain and response time (RT) were analyzed as behavioral metrics. The five behavioral data (HR, OP, FR, RR, RT) were homogeneous in variance (Bartlett's test) and followed a normal distribution (Shapiro–Wilk test). However, the SIR was homogeneous in variance but was not normally distributed. The four behavioral data types (HR, OP, FR, RR) were analyzed using a 3 × 2 analysis of variance (ANOVA) with the time (Block 1, Block 2 and Block 3) as a within-subject factor and the condition (FF, FB) as a between-subject factor (Bonferroni corrected). Since the SIRs did not follow a normal distribution, they were analyzed using the Friedman test instead of the 3 × 2 ANOVA. An independent-sample *t* test was used to test whether the total amount of rewards earned by the proposer and responder differed between the FF and FB conditions. Moreover, the difference in the PANAS score of each pair between the FF and FB conditions was also *t* tested.

#### fNIRS data: Interpersonal brain synchronization

Wavelet transform coherence (WTC), defined as the cross-correlation between two time series as a function of frequency and time [22], has often been used to measure the IBS of fNIRS data [17, 23, 24]. In this study, the WTC of the HbO time series between pairs was analyzed using the MATLAB package (http://noc.ac.uk/using-science/crosswavelet-wavelet-coherence) in the frequency band between 12.8 and 51.2 s (i.e. 0.02–0.08 Hz) that was sensitive to our task. The frequency band was chosen based on the original study [17], which enabled the removal of low- and high-frequency noise from the raw HbO time series so that no additional filtering was required. We did not perform any preprocessing on the oxy-Hb signals, such as bandpass filtering or detrending, referring to Cui et al. [23].

Each trial's HbO time series from Stages 2 to 4 (where interaction occurred between participants in the FF condition) were extracted as task data. Consequently, the task data's duration was up to 500 s per block. The average coherence values in this band were calculated for the resting and task blocks. The relative change in the mean coherence values from the resting state to the task block was used as a measure of the change in IBS between pairs [17, 23, 24]. Fisher's z-transformation was used to transform the mean coherence change for statistical tests. To find the channels where significant synchronization was observed during the task, a one-sample *t* test (*p* < 0.05, two-tailed, false discovery rate [FDR] corrected) was performed on the coherence changes of all channels.

Next, we performed an independent samples *t* test (*p* < 0.05, one-tailed, FDR corrected) to test whether the coherence change during the task differed between the FF and FB conditions. The *t*-values of each channel derived from these tests were used to generate the *t*-maps, which were smoothed using the spline method. Then, bivariate Pearson's correlations between the coherence change and the SIR score were calculated to examine the association between IBS and behavior, and the calculated correlation coefficients were applied to the uncorrelated test (*p* < 0.05, FDR corrected).

Finally, we examined whether the IBS during the task was due to social interaction rather than task-derived effects. Pseudo pairs were created by randomly shuffling pairs of participants under the same conditions. This process included the following constraints: (1) the pseudo pair did not consist of the actually paired participants and (2) the pseudo pair consisted of proposer and responder roles. The IBS values of the paired and pseudo paired participants were compared using permutation analysis ([11, 25]. We repeatedly generated 1000 pseudo pairs and compared their coherence changes with those of real pairs. The *p* values were FDR-corrected for multiple comparisons ([11, 25]. All the statistical analyses (i.e. ANOVA, Friedman test, correlation test and *t* test) were performed using MATLAB (MathWorks, Natick, MA).

## RESULTS

### Behavioral results

There was no significant main effect of HR, OP and FR on both time (HR: *p* = 0.629, *F* = 0.467, *η_p_^2^* = 0.014; OP: *p* = 0.859, *F* = 0.152, *η_p_^2^* = 0.0046; FR: *p* = 0.831, *F* = 0.186, *η_p_^2^* = 0.0056) and condition (HR: *p* = 0.256, *F* = 1.313, *η_p_^2^* = 0.0195; OP: *p* = 0.436, *F* = 0.614, *η_p_^2^* = 0.0092; FR: *p* = 0.615, *F* = 0.255, *η_p_^2^* = 0.0038). The RT of shared intentionality showed a significant main effect of condition, indicating that pairs judged each other's behavior more quickly in FB than in FF (*p* = 0.016, *F* = 6.09, *η_p_^2^* = 0.085). Furthermore, there was no interaction effect of condition and time on these measures. There were significant differences in the SIR and RR between the conditions (SIR, *p* = 0.003, *F* = 13.106, *η_p_^2^* = 0.191; RR: *p* = 0.047, *F* = 4.109, *η_p_^2^* = 0.0586). However, there was neither a significant main effect of time nor a condition × time interaction effect on these behavioral measures. There was no significant difference between the conditions for the total amount of game gains (*p* = 0.43, *d* = 0.33). However, there was a significant difference in gains between the proposer and responder in both conditions (FF: *p* = 0.0012, *d* = 0.99; FB: *p* = 0.0003, *d* = 1.65). There was no difference in the PANAS score of each pair between the conditions (positive: *t* = −1.80, *p* = 0.077, *d* = 0.52; negative: *t* = 0.49, *p* = 0.62 *d* = 0.14).

### fNIRS results

The results of the one-sample *t* test showed that coherence did not increase significantly for all channels in the frequency range of 0.02 to 0.08 Hz (Fig. 3) in either condition. The independent-samples *t* test showed that there was no significant difference between conditions in the coherence increases during the task. No significant correlations were found between increasing coherence and SIR, for all channels in either condition (Fig. 4).

The results of the random pair analysis for the task block identified that the IBS of the interacting (real) pair was significantly greater than that of the pseudo pair in both conditions (Fig. 5 and Table 2). In the FF condition, there were 11 channels in which the IBS of the interacting pair was greater than that of the pseudo pair (*P* < 0.05, FDR-corrected): channel (CH) 4 (right superior frontal gyrus [SFG.R]), CH5 (right middle frontal gyrus [MFG.R]), CH8 (SFG.R), CH9 (MFG.R), CH12 (SFG.R), CH16 (SFG.R), CH20 (right orbital superior frontal gyrus [ORBsup.R]), CH34 (right superior temporal gyrus [STG.R]), CH36 (right postcentral gyrus [PoCG.R]), CH37 (STG.R), CH46 (right precentral gyrus [PreCG.R]). In the FB condition, two channels had significantly greater IBS values in the interacting pair than in the pseudo-pair (*P* < 0.05, FDR-corrected): CH10 (MFG.R) and CH14 (left orbital superior frontal gyrus [ORBsup.L]). Contrarily, the results of the random pair analysis for the resting-state block showed no significant difference in the IBS between interacting and pseudo pairs for all channels. This result was the same for both the conditions.

**Table 2:** The channels where the IBS of the interacting pair was significantly greater than that of the pseudo pair. Channels (CHs), conditions and the anatomical regions associated with the CHs, *P* value, and FDR- corrected *P* value are listed

CH	Condition	Anatomical region	*P*	*P* (FDR-corrected)
4	FF	SFG.R	< 0.001	< 0.001
5	FF	MFG.R	< 0.001	< 0.001
8	FF	SFG.L	< 0.001	< 0.001
9	FF	MFG.L	< 0.001	< 0.001
12	FF	SFG.L	0.002	0.013143
16	FF	SFG.R	0.004	0.0184
20	FF	ORBsup.R	0.003	0.01725
34	FF	STG.R	< 0.001	< 0.001
36	FF	PoCG.R	0.004	0.0184
37	FF	STG.R	< 0.001	< 0.001
46	FF	PreCG.R	0.005	0.020909
10	FB	MFG.R	0.001	0.023
14	FB	ORBsup.L	0.001	0.023

Abbreviations: MFG, left middle frontal gyrus; SFG, superior frontal gyrus; SFGmed, superior frontal gyrus, medial; ORBmid, middle frontal gyrus, orbital; ORBsup, superior frontal gyrus, orbital; STG, superior temporal gyrus, PoCG, postcentral gyrus; R, right; L, left.

## DISCUSSION

### Behavioral results

Here, we examined the role of shared intentionality in FF and FB interactions, with a particular focus on the fairness and acceptance of reward distribution in acquainted pairs. In the following sections, we delve into the details of our results, discussing in sequence the replicated significant results, the replicated non-significant results and the findings that were not replicated.

**Figure 2:**
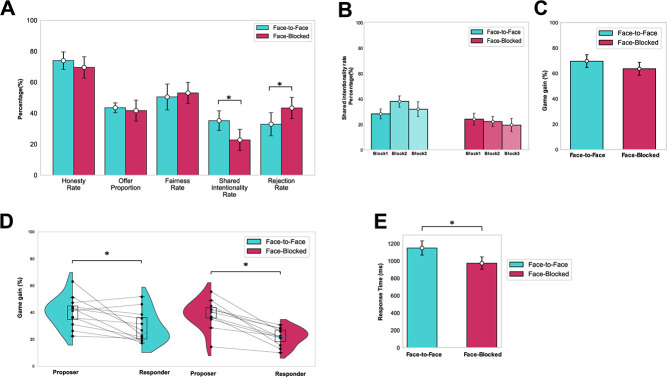
**Behavioral analysis results.** (A) Game measures. The SIR and RR were significantly different between the FF and FB conditions (*p* < 0.05), but the FR, OP, HR were not significantly different. Error bars indicate standard errors. (B) Time effect of SIR. There was no significant difference between the task blocks in either the FF or FB conditions. Error bars indicate standard errors. (C) Gains in the game of proposer and responder. There was no significant difference between the proposer and Responder’s gains between the conditions. (D) Total amount earned for each role. In both conditions, the gains were significantly higher for the proposer than for the responder (FF: *P* = 0.012; FB: *P* = 2.7 × 10^−4^). Black lines connect the values of the pairs that participated in the experiment together. (E) Response time of shared intentionality (ms) in each condition. The pairs judged each other’s behavior more quickly in FB than in FF (*p* = 0.016, *F* = 6.09, *η_p_^2^* = 0.085). Abbreviations: FF, face-to-face; FB, Face-blocked; FR, fairness rate; OP, offer proportion; HR, honesty rate; SIR, shared intentionality rate

First, we report the visual observation results indicated in Fig. 2A. The FR and the SIR was lower, while the RR was higher compared to the original study. More than 50% of trials were allocated to the fair condition, which might imply a fair or near-fair distribution. However, Fig. 2D displays significantly higher earnings for the proposer compared to the responder, suggesting a fair distribution was not effectively achieved. Thus, the lower FR and higher RR with SIR can be attributed to the unfairness of the distribution. These tendencies imply that closer social distance may have some influence on fair behavior.

Next, we discuss the replicated significant results. There were significant between-condition differences in the behavioral measures of SIR and RR (Fig. 2A). Previous studies have shown that social cues play an important role in building trusting relationships with others [26, 39]. In the FF condition, participants could communicate verbally and nonverbally (e.g. eye contact and facial expression) in real-time, which may have facilitated an increase in the SIR. Our results imply that the increased SIR between the pair and the responder's positive rating of the proposer's behavior made the offer more likely to be accepted.

Then, the replicated non-significant results are discussed. There were no significant between-condition differences in the behavioral measures (FR, OP and HR) associated with the proposer (Fig. 2A). In general, it is thought that proposers distribute rewards fairly based on two motives in ultimatum games: (1) an altruistic motive due to the social norm that rewards should be distributed fairly, and (2) a strategic motive to prevent Responders from rejecting proposals [27]. A previous study has shown that disclosure of information about the responder does not affect the proposer's offer and suggests that proposers distribute rewards with strategic rather than altruistic motives [40]. In the present study, the proposer's selfish behavior tended to be the same as in the previous study, even in acquainted pairs (Fig. 2A). Therefore, our results indicate a possibility that the FF condition or acquaintance with the partner might not significantly impact the Proposer's behavior in terms of fair reward distribution.

Lastly, the four earlier findings of the original study were not replicated. First, in contrast to the original study, we did not find a significant main effect of time on the SIR in the FB condition (Fig. 2). In the original study, participants in the FB condition did not meet their partners directly. They could only infer their partner's personality through their behavior during the task. Therefore, since trust in the partner depends on the partner's behavior during the task [39], it is considered that the SIR in this condition decreased over time. On the other hand, the participants in the FB condition of our study did not see their partner during the task, but they knew their identity and personality. Therefore, it can be presumed that expectations and predictions about the partner's behavior were made based on prior information ([28, 29]. Thus, it can be inferred that even in the non-FF condition, the behavior during the task did not significantly affect shared intentionality. Second, the total amount of money earned by the Proposer and the Responder was not significantly different between the conditions (Fig. 2C). Third, the results of this study had a lower SIR and higher RR than those of the previous study (Fig. 2A). Furthermore, it should be noted that the order of RT between conditions was reversed compared to the original study. In the original study, participants responded faster in the FF than to the FB condition, but in our study, participants responded faster in the FB condition (Fig. 2E). The original study interpreted their RT results that the FF compared to the FB interactions elicited greater shared intentionality between partners resulting in less rejection and more quick intention judgment, and consequently facilitated cooperation such that proposers were more likely to infer that responders would accept their offer and responders were more likely to believe that proposers told the truth [17]. On the other hand, although it is based on visual observation, our result exhibited a tendency for lower SIR and higher RR, which is opposite to those of the original study. These results suggest that pairs could effectively use external visible cues such as facial expressions in situations with higher shared intentionality. In contrast, when SIR is lower, as observed in our study compared to the original study, it might require more time to infer each other's intentions in the FF condition. Importantly, while the SIR value was indeed higher in the FF condition than the FB condition in our study, both were lower than those reported in the original study.

These results suggest that the pairs in this study may not have cooperated as well as those in the original study. Wu et al. [30] examined how social distance affects recipients' evaluations of unfair behavior. They reported that in a dictatorship game, participants did not react negatively to the unfair behavior of strangers but confirmed negative reactions to the unfair behavior of their friends. This result suggests that the lesser the social distance to the other person, the more likely they are to demand fair behavior from that person. Because the social distance between the pairs in this study was closer than in the original study, it is considered that the responder required the proposer to distribute the reward more fairly than when the partner was a stranger. Lastly, the RR in this study was higher than that in the original study, which may indicate that the inequitable distribution accepted by the responder in the previous study was rejected in this study. In addition, as shown in Fig. 2D, proposers had higher gains than responders, regardless of the condition. This suggests that proposers tended to pursue their own interests, while responders may have rejected offers and corrected the inequitable behavior. This could also explain the low SIR.

### Interpersonal brain synchronization

In the following discussion, we delve into our findings related to IBS in FF and FB interaction conditions. We explore why our results differ from the original study, focusing on the influence of social distance on participants' cooperative behavior and IBS. The specific brain regions associated with these interactions and their implications are discussed. Also, our study's strengths are addressed, including its novel exploration of social interaction's effect on IBS and the successful identification of pair-specific IBS regions. In addition, we acknowledge and discuss the limitations we encountered during our research, covering potential sampling bias, methodological challenges and interpretational issues. Finally, we look ahead to future directions of the current study.

**Figure 3:**
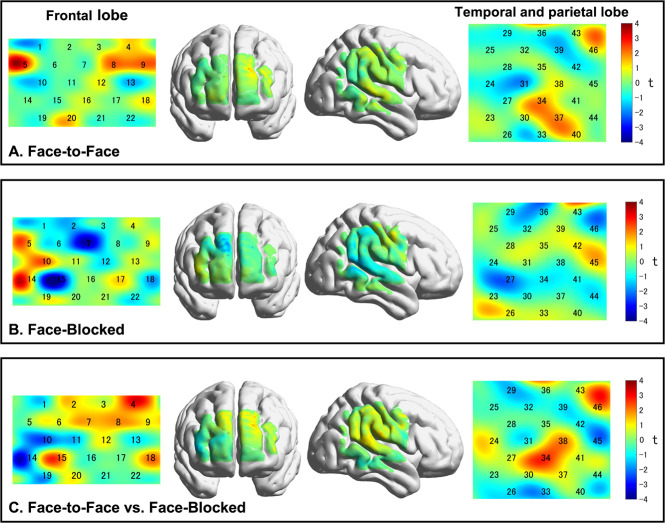
Comparison of the IBS between different conditions. (A, B) one-sample t-test for the increased coherence. In both conditions, the coherence increases in the task block from the resting state block were not significant for all channels (*p* > 0.05). (C) Independent sample t-test for the coherence increases between conditions. No conditional differences were found for all channels.

For IBS, the results of the original study were not replicated in our study. Here, we discuss the reasons for these differences from prior studies on IBS. In this study, there was no significant increase in coherence during the task in either the FF or FB conditions (Fig. 3A and B). Furthermore, there was no significant between-condition difference in coherence increase during the task (Fig. 3C). Previous research has shown that the lesser the social distance to the partner, the more cooperative the behavior and the greater the IBS [43].

Therefore, it is natural to expect the IBS to be greater than in previous studies because we decreased the social distance between the pairs. Here, it is important to clarify that this assumption is not a predefined hypothesis but rather on exploratory observation conducted after the commencement of the experiment. However, the results were contrary to our expectations, which could mean that the pairs who participated in this study did not cooperate as well as those in the previous studies. According to the behavioral analysis results, the SIR between pairs was lower, and the RR was higher in this study than in the previous study. One reason for this may be that it took a long time to come to a mutually compromised offer between the pairs because they were closer in social distance. Therefore, the IBS of acquainted pairs who did not act cooperatively may have been lower than that of stranger pairs who did cooperate.

Although the earlier IBS findings in the original study were not replicated in our acquainted pairs, these results support the idea of Gvirts and Perlmutter [16] that IBS is influenced by with whom we interact and how. The following points have been suggested from the results of this and the original studies: (1) If the pairs are not acquainted (i.e. the social distance between the pairs is great), FF interaction makes it easier for them to infer each other's intentions and states of mind and cooperate. (2) Even if the pairs are acquainted (i.e. the social distance between the pairs is small), FF interaction makes it easier for them to guess each other's intention and state of mind, as in the case of the stranger pairs. However, acquaintance promotes the feeling that the partner should behave fairly, preventing them from cooperating. In summary, the results suggest that feelings toward others and the process of building shared intentionality differ depending on the social distance between members of a pair.

**Figure 4:**
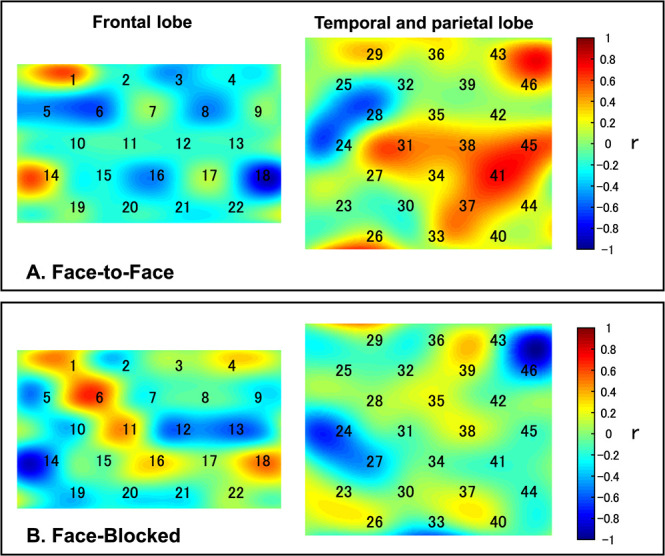
Correlations between the coherence increase and the SIR. Correlation coefficients between increased coherence and the SIR for all CHs are shown in the r-map. (A, B) There was no significant correlation between increased coherence and the SIR in either condition. Abbreviations: SIR, shared intentionality rate; CHs, channels

Furthermore, we performed random pair analysis to confirm whether the IBS during the task was specific to the interacting pair. Figure 4 shows that the IBS of the actual pairs was significantly greater than that of the pseudo pairs. In other words, the synchronization of brain activity was not due to the execution of the same tasks between two persons but to social interaction between pairs. Therefore, the channels (brain regions) where IBS is observed are specific to the interacting pair. Previous studies have also shown that the IBS of interacting pairs is significantly larger than that of pseudo pairs [11, 31].

**Figure 5:**
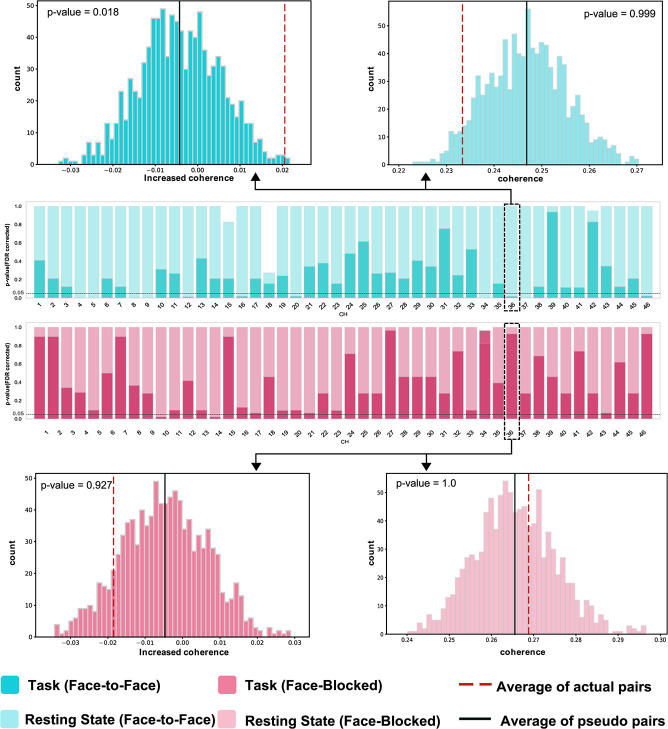
Results of the permutation test in random pair analysis (for the task and resting-state blocks). 11/46 channels indicated greater coherence in the real pair than in the pseudo pair for the task block, in the FF condition (*P* < 0.05, FDR corrected). In the FB condition, 2/46 channels were identified. There was no significant difference in coherence between interacting and pseudo pairs for all channels for the resting-state block. Abbreviations: FF, face-to-face; FB, face-blocked; FDR, false discovery rate

Additionally, the random pair analysis results for the resting-state block showed that the coherence of the interacting pairs was not significantly different from that of the pseudo pairs, which was the same for both conditions. This result suggested that the increase in IBS was not induced simply by being in the same space and time but only by social interaction. In addition, the brain regions where IBS in the real pairs was greater than that in the pseudo pairs were involved in social cognitive functions. In both conditions, pair-specific IBS was observed in the left and right SFG, MFG and ORBsup. This may be because the brain functions involved in executing the ultimatum game are common to both conditions. The dorsolateral prefrontal cortex (DLPFC) is involved in working memory functions that store opponent responses during repeated strategic games [27, 32] and in executive control functions that control selfish and altruistic behavior [33, 34].

The ORBsup is involved in learning from past experiences and predicting rewards. It is presumed that participants learned about their own and others' behavior and the associated consequences in the ultimatum game and engaged in trial and error of the optimal behavior to maximize their reward. Only in the FF condition, pair-specific IBS was observed in the STG.R, PreCG.R and PoCG.R. This may be because social cognitive functions were more active in the FF condition. Notably, the PreCG and PoCG are involved in cognitive empathy [35, 36]. In ultimatum games, participants need to predict the intentions and beliefs of their opponents by taking their opponents' point of view and control their selfish choices (e.g. unfair distribution and rejection as punishment for unfair distribution), and the IBS in the PreCG.R and PoCG.R could contribute to self-control. The STG is involved in the theory of mind, mentalizing and the ability to understand others’ mental states ([37, 38]; Carter et al., 2013). The STG, a constituent region of the TPJ, is known for integrating and processing social signals. In the FF condition, participants were able to observe their partner’s social signals nonverbally. This may allow the participants to infer their partner’s intentions and emotions and try to understand each other’s strategies.

In summary, the participants focused their attention on their partner, memorized their actions and outcomes and predicted the other person's actions based on their past experiences. It is believed that they were trying to achieve the goal of maximizing rewards through these behaviors. Furthermore, FF interaction allows participants to communicate nonverbally, making it easier for them to infer the other person's intentions and feelings because they can receive feedback from the other person on their own actions. We identified the key regions (STG.R, PreCG.R and PoCG.R) of the above-mentioned interaction in the FF condition using random pair analysis.

### Strengths of the current study

This study aimed to (1) explore if the results of Tang et al. [17] can be observed in acquainted pairs and (2) investigate whether IBS during the task could be influenced by social interaction. For the first objective, we found a similar trend in some behavioral data. However, the results differed from those of previous studies regarding low pairwise reliability, high RR and the lack of significant IBS. These results suggest that changing the nature of the interacting partners (changing the pairs from strangers to acquaintances, reducing the social distance) might cause changes in the participants’ feelings toward their partners during the task and in the process of building cooperation and shared intentionality.

The results of previous studies and our behavioral data revealed that the interaction setting (FF or FB) resulted in changes in the participants’ cooperative behavior. It was confirmed that social interaction-induced IBS is affected by the nature of the interacting partners. The results of the original study by Tang et al. [17] and the current study support the idea of Gvirts and Perlmutter [16] that IBS is influenced by with whom we interact and how. We did not identify any significant IBS, but we did find pair-specific IBS results using random pair analysis. We succeeded in identifying such pair-specific regions: left and right SFG, MFG and ORBsup, and the right STG, PreCG and PoCG. Furthermore, the coherence of these IBSs in the resting state did not significantly differ between the real and pseudo pairs, indicating that coherence was increased by the social interaction between the real pairs.

### Limitations of the current study and future direction

Despite the valuable insights gained from this study, there are several limitations that must be acknowledged. First, our sample was comprised of individuals who were acquaintances and colleagues from the same laboratory at the time of the study. Therefore, we could not control for the sample size and sampling bias (social distance). One of the important future tasks is to conduct a power analysis based on Tang et al. [17], estimate the required sample size and clarify the impact of these factors on the results. Besides, pre-registration was not conducted in this study. In future studies, incorporating the preregistration scheme is mandatory to enhance reproducibility and reliability.

Second, we did use ANOVA as the analysis method, following to the original study. However, there were the possibility that the measured variables may have some covariance (e.g. lower fairness rate may associate with higher RR. Applying MANCOVA instead of ANOVA could potentially be an effective approach to address this issue and should be considered in future research. However, while MANCOVA can control for confounding variables, interpreting its results can be challenging and may not be appropriate if certain assumptions are not met. Considering ways to handle covariates would be a significant issue in enhancing the reliability of the results.

Third, for the interpretation of IBS results, it cannot be said with certainty that the comparison between FF and FB is purely based on the opponent’s visualization because only the FF condition allowed participants to communicate not only by pressing the button but also verbally while the FB communicated only in pressing the button. To make this more confident, it is essential to use more comparable control conditions, such as the use of conversational tasks in both conditions (Drijvers & Holler, 2022; [24]). Also, since only Stages 2 and 3 included verbal communication only for the FF condition, it is worth analyzing IBS in other stages, except for these epochs for higher compatibility in the task between conditions.

Fourth, in this study, we did not directly remove signals related to artifacts, which is essential. However, the current study assumed that extracting the specific frequency band of the WTC analysis contributed to reducing the effect of the artifacts. Nonetheless, we cannot claim to have completely removed the presence of potential noise. As a future task, it is important to consider using equipment for directly measuring the physiological confounds and the motion artifacts (e.g. an accelerometer and short-distance channels) to examine their effect. Furthermore, the current study chose not to preprocess the data, a decision that diverged from the approach of the original study by Tang et al. [17]. Our choice was influenced by the precedent set by influential studies like [23], in which no preprocessing steps were undertaken. However, a recent study [44] states that preprocessing is a fundamental step of fNIRS data analysis. We acknowledge that these factors potentially affected our results and partially explain why some of the original study’s findings were not replicated. Replicating the original study at a more detailed level of preprocessing represents important areas for future work.

Fifth, our current study’s design and dataset do not permit a direct comparison with the original study conducted by [17] due to different probe settings and monetary units in representing the reward. Indeed, for more conclusive insights, it would be crucial to collect and directly compare data from both acquainted pairs and strangers. Such a comparative analysis would bolster the reliability of our current findings and yield a more comprehensive understanding of the disparities in inter-brain synchronization between these two groups. Pursuing this approach presents a worthwhile direction for future research endeavors.

Finally, the present study did not provide evidence of whether participants actually utilized visual cues. We were unable to collect behavioral data to confirm whether visual signals were effectively shared among participants. In future research, it will be important to verify whether information sharing between participants was conducted appropriately by utilizing methods, such as eye tracking or video recording. This improvement will provide further insight into the social interaction of dyads.

Taking these constraints into consideration, our study contributes to the field of social neuroscience by providing new perspectives on the influence of social interactions on inter-brain synchronization (IBS). Contrary to the original study's findings, our results did not demonstrate any task-induced IBS increase or correlation with cooperative behavior. Instead, our random pair analysis revealed significant pair-specific IBS only under the task condition, observable in the left and right SFG, MFG, ORBsup and the right STG, PreCG and PoCG. These findings tentatively suggest that FF interaction in acquainted pairs might not increase IBS, hinting at the possibility that the nature of IBS could be influenced by whom we interact with and how. As such, we cautiously propose that the current study might offer some preliminary insights into the complex dynamics of social distance manipulation on IBS.

## STUDY FUNDING

This work was supported by JSPS KAKENHI Grant Number JP20K11963.

## CONFLICT OF INTEREST STATEMENT

The authors report no conflict of interest.

## DATA AVAILABILITY

The data underlying this article will be shared on reasonable request to the corresponding author.

## Supplementary Material

Web_Material_kvad007
