# Supplementary material for: Interpersonal brain synchronization during face-to-face economic exchange between acquainted dyads
Source: Oxf Open Neurosci. 2023 Jun 21;2:kvad007. doi: 10.1093/oons/kvad007 (PMC10913825; doi:10.1093/oons/kvad007)
Supplement: Web_Material_kvad007 [file Web_Material_kvad007.zip › Reviewers comments - Supplementary Material - OXFNSC-2022-017.R2 .docx]

**REVIEWER COMMENTS**

**OXFNSC-2022-017.R2- Interpersonal brain synchronization during face-to-face economic exchange between acquainted dyads**

**Original submission - OXFNSC-2022-017**

**Reviewer 1**

Comments to Author

The authors examined the association between Interpersonal Brain Synchronization (IBS) and dyadic economic exchange. The study aimed at replicating and generalizing the main findings of Tang et al. (2016). Specifically, the original study was conducted with dyadic pairs of strangers. The current study replayed the same experimental protocol of the adjusted ultimatum game with dyadic pairs of aquatints. During the game, one of the participants played the role of “provider” who offered a deal and the other played the role of “responder” who decided to accept or reject the offer. In addition, before making the decision, the dyad was asked to make judgments about each other.

The authors managed to replicate the findings of lower reject rate and enhancement of positive mutual judgment between the two participants, when they perform the task face-to-face, in contrast to face-blocked. Unexpectedly, the authors did not observe significant changes in IBS related to the task, in contrast to the resting-state, nor significant differences of IBS between face-to-face and face-blocked conditions, as reported in the original study. The authors discussed that such negative results could be a consequence of closer social distance of the participants in current study that might lead to a lower cooperativeness of them, as they might require more fairness in the deal. Moreover, the author conducted additional analysis of contrasting IBS between real interacting dyads and random dyads. A higher task-related IBS was found in the former than the latter, but no differences of resting-state IBS. These results suggested that economic exchange is associated with higher IBS and the effect might be modulated by the relationship of the interacting partners.

I read both the manuscript and the original study; and I appreciated that the authors carefully replayed the experimental and analytical protocol. The motivates, hypothesis, methods and results of the study were legibly presented. I am especially glad to see that the author conducted the control analysis using random pairs that provides more insight to the research question. The authors also provided a possible explanation for the null results of IBS in relation to social distance. I think this is a very interesting avenue to follow up, but the interpretation of results is not quite clear to me. However, I believe the author could help me better understand their points in a revision. I will elaborate my questions in the comments below. I understand that second-person neuroscience is a new and challenging field. It is important to make replication studies and negative results visible to the audience. I think this study was fairly conducted, though I have serval major comments on the original experimental design and interpretation of the results. I hope the author would be given a chance to elaborate more on their reflection on the original study, interpretation of the current results based on existing literature and/or provide more empirical evidence for their argument.

Major comments
1. I have a concern regarding the original experiment design. As the participants were doing very different things in FF (talking) and FB (pressing buttons), the difference of IBS between the conditions could hardly be confirmed to be related to the visualization of the partner. The authors also followed the interpretation as in the original study, such as “In the FF condition, participants could communicate nonverbal in real-time, which may have facilitated an increase in the SIR.”. It is worth to note that in the FF condition, the participant can talk to each other actual. And on page 29, the author claimed “From the results of this and the original studies, the following points have been clarified …” I would suggest the authors to be more cautious when interpreting FF vs. FB results, especially with the respect to IBS. Moreover, a few hyperscanning studies using conversational task (e.g. Drijvers & Holler, 2022; Jiang et al., 2012) required the participants to talk face-to-face or face-block (or back-to-back). It might be worth to consider using more comparable control conditions.

2. The author claimed, “In summary, the results suggest that feelings toward others and the process of building shared intentionality differ depending on the social distance between members of a pair.” Can they rule out other possibilities that might leading to negative results comparing to the original study. Would the authors consider the negative results was due to sampling noise or acquaintance or both? For instance, 1) Sampling bias, the current sample tend to have less cooperative behavior (lower SIR and higher RR), which might be independent from social distance. 2) Sample size, the current sample is only as half as the original study, but some of the negative results (e.g. Figure 2C, Figure 3) seems showing similar patterns as the original findings. Besides, the current study also managed to reproduce most of the behavioral findings. Regarding the replication procedure, I am wondering if the author did a power analysis based on Tang et al. (2016). The author might want to specify how they decided the sample size and what they expected if they doubled the sample size.

3. The authors mentioned two possible reasons for the higher RR as compared to the original study. “However, we could not determine whether the Responder's motivation to reject the offer was altruistic (i.e., to correct the inequitable behavior) or selfish (i.e., to increase their reward).” In my humble opinion, a lower SIR and higher RR seems more likely driven by demands of fairness. If the participant just wanted to maximize the reward, they could accept all the deal no matter to what extent they trust or willing to cooperate with each other, then it should lead to a low RR and low SIR. Moreover, a low RR and low RR does not necessarily link with selfishness. It could be driven by an altruistic motivate as well. For instance, for being pilot, we sometimes might still accept unfair offer, even though we are not willing to. Maybe the authors could use a more specified term for “to correct the inequitable behavior”, instead of the broad term “altruistic”.

Minor comments
4. It is always encouraged to preregister especially for replication studies. If the authors follow that procedure, it would be good to mention it in the manuscript. If not, it would be good to consider such approach for future studies.

5. I understand that the authors were following the analytical protocol of the original study. I have a concern about the ANOVA analyses, as the measures may be to some extend covaried. For instance, lower fairness rate may associate with higher rejection rate. Therefore, if I were conducting the study, I may consider using MANCOVA instead of ANOVA.

6. The current study reported a lower SIR than that of the original study. I am wondering if the author also replicated the finding of the reaction time of during stage 4 (Figure 2B of Tang et al., 2016). The reaction time might provide more insight of certainty of the participants’ judgment.

7. In the introduction, the author briefly touched Gvirts & Perlmutter (2020). Perhaps it would be helpful if the author could provide simple examples of the three factors mentioned in Gvirts & Perlmutter (2020) for readers who are not familiar with that paper.

8. In the last paragraph of introduction, the authors mentioned, “One of the pitfalls of hyperscanning is that …”. Yes, this is a problem recognized in this field and discussed in the literature. Would it be more appropriate to add some reference to this point?

9. How is the measure “gain” defined?

10. In the discussion, the authors noted, “we expected the IBS to be greater than in previous studies because we decreased the social distance between the pairs.”. This hypothesis was not mentioned in the introduction.

11. The author wrote, “One reason for this may be that it took a long time to come to a mutually compromised offer between the pairs because they were closer in social distance.” Is this statement based on existing literature? If so, please add a reference. Does the statement mean that people tend to be more pilot when interacting with strangers, so they are more likely to accept an offer? In that case, both the RR and SIR would be low, isn’t it?

12. “Gvirts et al. (2020)” is a two-author paper and should be cited as “Gvirts & Perlmutter (2020)”.

Reference
Drijvers, L., & Holler, J. (2022). Face-to-face spatial orientation fine-tunes the brain for neurocognitive processing in conversation. IScience, 28.
Gvirts, H. Z., & Perlmutter, R. (2020). What Guides Us to Neurally and Behaviorally Align With Anyone Specific? A Neurobiological Model Based on fNIRS Hyperscanning Studies. The Neuroscientist, 26(2), 108–116. https://doi.org/10.1177/1073858419861912
Jiang, J., Dai, B., Peng, D., Zhu, C., Liu, L., & Lu, C. (2012). Neural Synchronization during Face-to-Face Communication. Journal of Neuroscience, 32(45), Article 45. https://doi.org/10.1523/JNEUROSCI.2926-12.2012
Tang, H., Mai, X., Wang, S., Zhu, C., Krueger, F., & Liu, C. (2016). Interpersonal brain synchronization in the right temporo-parietal junction during face-to-face economic exchange. Social Cognitive and Affective Neuroscience, 11(1), 23–32. https://doi.org/10.1093/scan/nsv092

**Reviewer 2**

Comments to Author

The current manuscript aimed at replicating previous findings by Tang et al. (2016). In the original study, the ultimatum game and functional near-infrared spectroscopy (fNIRS) hyperscanning were used to investigate how face-to-face interactions modulate interpersonal brain synchronization during economic exchange in pairs of strangers. In the current manuscript, the authors aimed at extending the original findings to pairs of acquaintances and, additionally, test the hypothesis that interpersonal brain synchronization depends on social interaction (i.e. the person who we interact with). In particular, both interpersonal brain synchronization and several behavioral measures in relation to the ultimatum game were compared between conditions (i.e. face-to-face and face-blocked) and across time (i.e. experiment blocks). The authors replicated some of the original findings: the shared intentionality rate and the rejection rate differed significantly across conditions and no significant differences across conditions were observed in the remaining behavioral measures. Additionally, the authors showed that interpersonal brain synchrony is significantly higher in interacting pairs in comparison with pseudo-pairs in both conditions.

The study presents interesting results and I particularly appreciate the choice of replicating and extending the results of a previous study and the choice of conducting the pseudo-pairs analysis to exclude potential confounding factors. Nonetheless, I have some major concerns regarding the pre-processing of the fNIRS data, the experiment power and the interpretation of the results.

1) In the manuscript, there is no description of any pre-processing technique for the fNIRS data. Pre-processing is a fundamental part of data analysis and the authors should describe accurately which pre-processing steps were taken (see Yücel et al. 2021. Best practices for fNIRS publications, Neurophotonics). Was the data corrected for motion? Was the data visually inspected for bad channels and for physiological artifacts? If not, how did the authors account for these potential noise sources?

Similarly, no information is provided about the software used for statistical analyses (i.e. ANOVAs, t-tests and Friedman test). Could the authors add this information?

2) In the original study by Tang et al. (2016), the fNIRS analysis was conducted on two specific regions: right TPJ as region of interest and right DLPFC as control region. Is there a specific reason for which the authors of the current manuscript conducted the fNIRS analysis including more extended frontal and temporal regions and did not include a control region?
If the aim of the study was to replicate the findings of Tang et al. (2016), why were additional regions included?

3) The original study by Tang et al. (2016) had a sample size of 202 participants. The current study used a sample size of 48 participants and the reported effect sizes are quite low. My concern is that the differences in the results obtained may depend on the sample size. How did the authors ensure that the current study was not underpowered? Was a power analysis conducted? Alternatively, I would suggest to mention this in the discussion as a limitation and to add the effect sizes of the fNIRS results.

4) The structure of the discussion section (4.1) is somewhat confusing to me. Results, which were significantly tested, and conclusions coming from visual inspections are intermixed throughout the section. In my opinion, in this way it is not clear to the reader, what was statistically evaluated and what was not. I would suggest to separate these observations in different paragraphs: visual inspection of the results, replicated significant and non-significant results and non-replicated results.

With regard to the visual inspection of the results (i.e. results which were not statistically evaluated), I refer specifically to the comparison between the current and the original study. No statistically test was used to evaluate the differences between studies, but strong conclusions are drawn (see lines 55-58 of page 24 and lines 43-46 of page 25). I think it is very relevant to compare the results from the previous and the current experiment, but caution should be taken when reporting the observed similarities and differences. These can be speculations and possible interpretations by the authors, but no strong conclusions should be drawn. Alternatively, the authors could statistically test the differences in the results obtained in the original study and in the current manuscript.

5) At the beginning of the discussion on behavioral results, it is reported that the Proposer tended to distribute fairly rewards and the Responder tended to reject the offer (lines 34-40, page 24), with a reference to figure 2A. The figure shows the fairness rate to be between 50% and 60% and the rejection rate between 30% and 40%. Are these considered high values in the context of the ultimatum game? Are these values similar to the ones obtained in the original study?

6) In the results, the authors report that gain percentage between Proposer and Responder was significant in both conditions (FF and FB) and this is illustrated in a nice graph (figure 2D). Surprisingly, this finding was not mentioned in the discussion. Is there a specific reason for this? I think discussing this finding is extremely relevant and could give further insights on the authors’ hypotheses and conclusions.

7) In the manuscript, the face-to-face condition is described as a condition in which participants can use non-verbal behavior (eye-gaze and hand gestures). Considering that the availability of non-verbal visual cues is mentioned in the interpretation of the results, I am concerned about the actual visibility of participants’ visual communicative signals.

The experiment set-up is nicely illustrated in figure 1A (left). Considering the monitors in front of the participants, I am wondering if participants could actually have a good visibility of their co-participants. I am particularly concerned that hand gestures would not be visible as the gesture space is covered by the monitor and it is hard to judge the visibility of the co-participant’s face from the figure. How did the authors ensure the visibility of the co-participants’ face and that this was similar across pairs? Did the authors record additional data (e.g. eye gaze, video recordings) which could be used to evaluate if the participants were indeed attending to their co-participant’s visual communicative signals?

Minor details:
• In figure 2C, the y-axis reports the ‘gain percentage’, while the plot likely shows the total amount of money earned by the Proposer and the Responder (as described in line 10-12 on page 27). Could the authors correct the y-axis label?
• In figure 2D, the gain percentage is significantly different between Proposer and Responder in both conditions, but there are no asterisks indicating the significant relationships. However, asterisks are used to illustrate significant differences in figure 2A. I think the figure would be clearer if significant differences were indicated in the same way throughout the figure.
• In the results section on behavioral data (2.4.1, page 17, lines 37-39), it seems that the shared intentionality rate (SIR) and the positive and negative affect schedule (PNAS) are not considered as behavioral variables. Could the authors clarify this in the manuscript, to avoid causing confusion to the reader?

**Decision letter - OXFNSC-2022-017**

09-Feb-2023

Dear Dr. Hiwa,

Manuscript ID OXFNSC-2022-017 entitled "Interpersonal brain synchronization during face-to-face economic exchange between acquainted dyads" which you submitted to the Oxford Open Neuroscience, has been reviewed.  The comments of the reviewer(s) are included at the bottom of this letter.

Please note that this journal operates with transparent peer review. This means that if your submission is accepted for publication, the full peer review history of your article will publish online alongside your article. This includes reviewer comments, editor decision letters, and your author responses.

The reviewer(s) have recommended publication, but also suggest some major revisions to your manuscript.  Therefore, I invite you to respond to the reviewer(s)' comments and revise your manuscript.

To revise your manuscript, log into https://mc.manuscriptcentral.com/oxfnsc and enter your Author Centre, where you will find your manuscript title listed under "Manuscripts with Decisions."  Under "Actions," click on "Create a Revision."  Your manuscript number has been appended to denote a revision.

You may also click the below link to start the revision process (or continue the process if you have already started your revision) for your manuscript. If you use the below link you will not be required to login to ScholarOne Manuscripts.

*** PLEASE NOTE: This is a two-step process. After clicking on the link, you will be directed to a webpage to confirm. ***

https://mc.manuscriptcentral.com/oxfnsc?URL_MASK=cba0df73e14d4c13887868253efa80c8

You will be unable to make your revisions on the originally submitted version of the manuscript.  Instead, revise your manuscript using a word processing program and save it on your computer.  Please also highlight the changes to your manuscript within the document by using the track changes mode in MS Word or by using bold or colored text.

Once the revised manuscript is prepared, you can upload it and submit it through your Author Centre.

When submitting your revised manuscript, you will be able to respond to the comments made by the reviewer(s) in the space provided.  You can use this space to document any changes you make to the original manuscript.  In order to expedite the processing of the revised manuscript, please be as specific as possible in your response to the reviewer(s).

IMPORTANT:  Your original files are available to you when you upload your revised manuscript.  Please delete any redundant files before completing the submission.

Because we are trying to facilitate timely publication of manuscripts submitted to the Oxford Open Neuroscience, your revised manuscript should be uploaded as soon as possible.  If it is not possible for you to submit your revision in a reasonable amount of time, we may have to consider your paper as a new submission.

Once again, thank you for submitting your manuscript to the Oxford Open Neuroscience and I look forward to receiving your revision.

Sincerely,
Professor Sam Gilbert
Senior Editor, Oxford Open Neuroscience
sam.gilbert@ucl.ac.uk

Reviewer: 1

Comments to the Author
The authors examined the association between Interpersonal Brain Synchronization (IBS) and dyadic economic exchange. The study aimed at replicating and generalizing the main findings of Tang et al. (2016). Specifically, the original study was conducted with dyadic pairs of strangers. The current study replayed the same experimental protocol of the adjusted ultimatum game with dyadic pairs of aquatints. During the game, one of the participants played the role of “provider” who offered a deal and the other played the role of “responder” who decided to accept or reject the offer. In addition, before making the decision, the dyad was asked to make judgments about each other.
The authors managed to replicate the findings of lower reject rate and enhancement of positive mutual judgment between the two participants, when they perform the task face-to-face, in contrast to face-blocked. Unexpectedly, the authors did not observe significant changes in IBS related to the task, in contrast to the resting-state, nor significant differences of IBS between face-to-face and face-blocked conditions, as reported in the original study. The authors discussed that such negative results could be a consequence of closer social distance of the participants in current study that might lead to a lower cooperativeness of them, as they might require more fairness in the deal. Moreover, the author conducted additional analysis of contrasting IBS between real interacting dyads and random dyads. A higher task-related IBS was found in the former than the latter, but no differences of resting-state IBS. These results suggested that economic exchange is associated with higher IBS and the effect might be modulated by the relationship of the interacting partners.

I read both the manuscript and the original study; and I appreciated that the authors carefully replayed the experimental and analytical protocol. The motivates, hypothesis, methods and results of the study were legibly presented. I am especially glad to see that the author conducted the control analysis using random pairs that provides more insight to the research question. The authors also provided a possible explanation for the null results of IBS in relation to social distance. I think this is a very interesting avenue to follow up, but the interpretation of results is not quite clear to me. However, I believe the author could help me better understand their points in a revision. I will elaborate my questions in the comments below. I understand that second-person neuroscience is a new and challenging field. It is important to make replication studies and negative results visible to the audience. I think this study was fairly conducted, though I have serval major comments on the original experimental design and interpretation of the results. I hope the author would be given a chance to elaborate more on their reflection on the original study, interpretation of the current results based on existing literature and/or provide more empirical evidence for their argument.

Major comments
1. I have a concern regarding the original experiment design. As the participants were doing very different things in FF (talking) and FB (pressing buttons), the difference of IBS between the conditions could hardly be confirmed to be related to the visualization of the partner. The authors also followed the interpretation as in the original study, such as “In the FF condition, participants could communicate nonverbal in real-time, which may have facilitated an increase in the SIR.”. It is worth to note that in the FF condition, the participant can talk to each other actual. And on page 29, the author claimed “From the results of this and the original studies, the following points have been clarified …” I would suggest the authors to be more cautious when interpreting FF vs. FB results, especially with the respect to IBS. Moreover, a few hyperscanning studies using conversational task (e.g. Drijvers & Holler, 2022; Jiang et al., 2012) required the participants to talk face-to-face or face-block (or back-to-back). It might be worth to consider using more comparable control conditions.
2. The author claimed, “In summary, the results suggest that feelings toward others and the process of building shared intentionality differ depending on the social distance between members of a pair.” Can they rule out other possibilities that might leading to negative results comparing to the original study. Would the authors consider the negative results was due to sampling noise or acquaintance or both? For instance, 1) Sampling bias, the current sample tend to have less cooperative behavior (lower SIR and higher RR), which might be independent from social distance. 2) Sample size, the current sample is only as half as the original study, but some of the negative results (e.g. Figure 2C, Figure 3) seems showing similar patterns as the original findings. Besides, the current study also managed to reproduce most of the behavioral findings. Regarding the replication procedure, I am wondering if the author did a power analysis based on Tang et al. (2016). The author might want to specify how they decided the sample size and what they expected if they doubled the sample size.
3. The authors mentioned two possible reasons for the higher RR as compared to the original study. “However, we could not determine whether the Responder's motivation to reject the offer was altruistic (i.e., to correct the inequitable behavior) or selfish (i.e., to increase their reward).” In my humble opinion, a lower SIR and higher RR seems more likely driven by demands of fairness. If the participant just wanted to maximize the reward, they could accept all the deal no matter to what extent they trust or willing to cooperate with each other, then it should lead to a low RR and low SIR. Moreover, a low RR and low RR does not necessarily link with selfishness. It could be driven by an altruistic motivate as well. For instance, for being pilot, we sometimes might still accept unfair offer, even though we are not willing to. Maybe the authors could use a more specified term for “to correct the inequitable behavior”, instead of the broad term “altruistic”.

Minor comments
4. It is always encouraged to preregister especially for replication studies. If the authors follow that procedure, it would be good to mention it in the manuscript. If not, it would be good to consider such approach for future studies.
5. I understand that the authors were following the analytical protocol of the original study. I have a concern about the ANOVA analyses, as the measures may be to some extend covaried. For instance, lower fairness rate may associate with higher rejection rate. Therefore, if I were conducting the study, I may consider using MANCOVA instead of ANOVA.
6. The current study reported a lower SIR than that of the original study. I am wondering if the author also replicated the finding of the reaction time of during stage 4 (Figure 2B of Tang et al., 2016). The reaction time might provide more insight of certainty of the participants’ judgment.
7. In the introduction, the author briefly touched Gvirts & Perlmutter (2020). Perhaps it would be helpful if the author could provide simple examples of the three factors mentioned in Gvirts & Perlmutter (2020) for readers who are not familiar with that paper.
8. In the last paragraph of introduction, the authors mentioned, “One of the pitfalls of hyperscanning is that …”. Yes, this is a problem recognized in this field and discussed in the literature. Would it be more appropriate to add some reference to this point?
9. How is the measure “gain” defined?
10. In the discussion, the authors noted, “we expected the IBS to be greater than in previous studies because we decreased the social distance between the pairs.”. This hypothesis was not mentioned in the introduction.
11. The author wrote, “One reason for this may be that it took a long time to come to a mutually compromised offer between the pairs because they were closer in social distance.” Is this statement based on existing literature? If so, please add a reference. Does the statement mean that people tend to be more pilot when interacting with strangers, so they are more likely to accept an offer? In that case, both the RR and SIR would be low, isn’t it?
12. “Gvirts et al. (2020)” is a two-author paper and should be cited as “Gvirts & Perlmutter (2020)”.

Reference
Drijvers, L., & Holler, J. (2022). Face-to-face spatial orientation fine-tunes the brain for neurocognitive processing in conversation. IScience, 28.
Gvirts, H. Z., & Perlmutter, R. (2020). What Guides Us to Neurally and Behaviorally Align With Anyone Specific? A Neurobiological Model Based on fNIRS Hyperscanning Studies. The Neuroscientist, 26(2), 108–116. https://doi.org/10.1177/1073858419861912
Jiang, J., Dai, B., Peng, D., Zhu, C., Liu, L., & Lu, C. (2012). Neural Synchronization during Face-to-Face Communication. Journal of Neuroscience, 32(45), Article 45. https://doi.org/10.1523/JNEUROSCI.2926-12.2012
Tang, H., Mai, X., Wang, S., Zhu, C., Krueger, F., & Liu, C. (2016). Interpersonal brain synchronization in the right temporo-parietal junction during face-to-face economic exchange. Social Cognitive and Affective Neuroscience, 11(1), 23–32. https://doi.org/10.1093/scan/nsv092

Reviewer: 2

Comments to the Author
The current manuscript aimed at replicating previous findings by Tang et al. (2016). In the original study, the ultimatum game and functional near-infrared spectroscopy (fNIRS) hyperscanning were used to investigate how face-to-face interactions modulate interpersonal brain synchronization during economic exchange in pairs of strangers. In the current manuscript, the authors aimed at extending the original findings to pairs of acquaintances and, additionally, test the hypothesis that interpersonal brain synchronization depends on social interaction (i.e. the person who we interact with). In particular, both interpersonal brain synchronization and several behavioral measures in relation to the ultimatum game were compared between conditions (i.e. face-to-face and face-blocked) and across time (i.e. experiment blocks). The authors replicated some of the original findings: the shared intentionality rate and the rejection rate differed significantly across conditions and no significant differences across conditions were observed in the remaining behavioral measures. Additionally, the authors showed that interpersonal brain synchrony is significantly higher in interacting pairs in comparison with pseudo-pairs in both conditions.

The study presents interesting results and I particularly appreciate the choice of replicating and extending the results of a previous study and the choice of conducting the pseudo-pairs analysis to exclude potential confounding factors. Nonetheless, I have some major concerns regarding the pre-processing of the fNIRS data, the experiment power and the interpretation of the results.

1) In the manuscript, there is no description of any pre-processing technique for the fNIRS data. Pre-processing is a fundamental part of data analysis and the authors should describe accurately which pre-processing steps were taken (see Yücel et al. 2021. Best practices for fNIRS publications, Neurophotonics). Was the data corrected for motion? Was the data visually inspected for bad channels and for physiological artifacts? If not, how did the authors account for these potential noise sources?

Similarly, no information is provided about the software used for statistical analyses (i.e. ANOVAs, t-tests and Friedman test). Could the authors add this information?

2) In the original study by Tang et al. (2016), the fNIRS analysis was conducted on two specific regions: right TPJ as region of interest and right DLPFC as control region. Is there a specific reason for which the authors of the current manuscript conducted the fNIRS analysis including more extended frontal and temporal regions and did not include a control region?

If the aim of the study was to replicate the findings of Tang et al. (2016), why were additional regions included?

3) The original study by Tang et al. (2016) had a sample size of 202 participants. The current study used a sample size of 48 participants and the reported effect sizes are quite low. My concern is that the differences in the results obtained may depend on the sample size. How did the authors ensure that the current study was not underpowered? Was a power analysis conducted? Alternatively, I would suggest to mention this in the discussion as a limitation and to add the effect sizes of the fNIRS results.
   
4) The structure of the discussion section (4.1) is somewhat confusing to me. Results, which were significantly tested, and conclusions coming from visual inspections are intermixed throughout the section. In my opinion, in this way it is not clear to the reader, what was statistically evaluated and what was not. I would suggest to separate these observations in different paragraphs: visual inspection of the results, replicated significant and non-significant results and non-replicated results.

With regard to the visual inspection of the results (i.e. results which were not statistically evaluated), I refer specifically to the comparison between the current and the original study. No statistically test was used to evaluate the differences between studies, but strong conclusions are drawn (see lines 55-58 of page 24 and lines 43-46 of page 25). I think it is very relevant to compare the results from the previous and the current experiment, but caution should be taken when reporting the observed similarities and differences. These can be speculations and possible interpretations by the authors, but no strong conclusions should be drawn. Alternatively, the authors could statistically test the differences in the results obtained in the original study and in the current manuscript.

5) At the beginning of the discussion on behavioral results, it is reported that the Proposer tended to distribute fairly rewards and the Responder tended to reject the offer (lines 34-40, page 24), with a reference to figure 2A. The figure shows the fairness rate to be between 50% and 60% and the rejection rate between 30% and 40%. Are these considered high values in the context of the ultimatum game? Are these values similar to the ones obtained in the original study?

6) In the results, the authors report that gain percentage between Proposer and Responder was significant in both conditions (FF and FB) and this is illustrated in a nice graph (figure 2D). Surprisingly, this finding was not mentioned in the discussion. Is there a specific reason for this? I think discussing this finding is extremely relevant and could give further insights on the authors’ hypotheses and conclusions.

7) In the manuscript, the face-to-face condition is described as a condition in which participants can use non-verbal behavior (eye-gaze and hand gestures). Considering that the availability of non-verbal visual cues is mentioned in the interpretation of the results, I am concerned about the actual visibility of participants’ visual communicative signals.

The experiment set-up is nicely illustrated in figure 1A (left). Considering the monitors in front of the participants, I am wondering if participants could actually have a good visibility of their co-participants. I am particularly concerned that hand gestures would not be visible as the gesture space is covered by the monitor and it is hard to judge the visibility of the co-participant’s face from the figure. How did the authors ensure the visibility of the co-participants’ face and that this was similar across pairs? Did the authors record additional data (e.g. eye gaze, video recordings) which could be used to evaluate if the participants were indeed attending to their co-participant’s visual communicative signals?  

Minor details:
• In figure 2C, the y-axis reports the ‘gain percentage’, while the plot likely shows the total amount of money earned by the Proposer and the Responder (as described in line 10-12 on page 27). Could the authors correct the y-axis label?
• In figure 2D, the gain percentage is significantly different between Proposer and Responder in both conditions, but there are no asterisks indicating the significant relationships. However, asterisks are used to illustrate significant differences in figure 2A. I think the figure would be clearer if significant differences were indicated in the same way throughout the figure.
• In the results section on behavioral data (2.4.1, page 17, lines 37-39), it seems that the shared intentionality rate (SIR) and the positive and negative affect schedule (PNAS) are not considered as behavioral variables. Could the authors clarify this in the manuscript, to avoid causing confusion to the reader?

Associate Editor
Comments to the Author:
(There are no comments.)

**Author response**

We highly appreciate the insightful and thoughtful comments raised by the reviewers to improve the quality of this manuscript. We were encouraged by the positive feedback from the reviewers and have addressed each comment carefully, making appropriate amendments based on their suggestions. We also provided a point-by-point response to the reviewer’s comments. In addition, we conducted additional analyses as suggested by the reviewers. The corrections in the revised manuscript were highlighted in yellow to facilitate the reviewers' evaluation.

[To Reviewer 1]
1. I have a concern regarding the original experiment design. As the participants were doing very different things in FF (talking) and FB (pressing buttons), the difference of IBS between the conditions could hardly be confirmed to be related to the visualization of the partner. The authors also followed the interpretation as in the original study, such as “In the FF condition, participants could communicate nonverbal in real-time, which may have facilitated an increase in the SIR.”. It is worth to note that in the FF condition, the participant can talk to each other actual. And on page 29, the author claimed “From the results of this and the original studies, the following points have been clarified …” I would suggest the authors to be more cautious when interpreting FF vs. FB results, especially with the respect to IBS. Moreover, a few hyperscanning studies using conversational task (e.g. Drijvers & Holler, 2022; Jiang et al., 2012) required the participants to talk face-to-face or face-block (or back-to-back). It might be worth to consider using more comparable control conditions.
Drijvers, L., & Holler, J. (2022). Face-to-face spatial orientation fine-tunes the brain for neurocognitive processing in conversation. iScience, 28.
Jiang, J., Dai, B., Peng, D., Zhu, C., Liu, L., & Lu, C. (2012). Neural Synchronization during Face-to-Face Communication. Journal of Neuroscience, 32(45), Article 45. https://doi.org/10.1523/JNEUROSCI.2926-12.2012
RESPONSE: Thank you for your insightful comments. In the FF condition, Proposer was instructed to verbally communicate the total amount to the Responder, which they set themselves in stage 2, and the transfer amount to the Responder in stage 3. Therefore, in the FF condition, Proposer communicated verbally. In addition, each response was recorded via button presses (added in Lines: 142-144). On the other hand, in the FB condition, there was no verbal conversation. Thus, as you pointed out, it cannot be said with certainty that the comparison between FF and FB is purely based on the opponent's visualization. To make this more confident, it is essential to use more comparable control conditions, considering the two papers you recommended as references. We have added these points as limitations and future directions (Lines: 555-561) and toned down ‘clarified’ to ‘suggested’ (Lines 444-445).

2. The author claimed, “In summary, the results suggest that feelings toward others and the process of building shared intentionality differ depending on the social distance between members of a pair.” Can they rule out other possibilities that might leading to negative results comparing to the original study. Would the authors consider the negative results was due to sampling noise or acquaintance or both? For instance, 1) Sampling bias, the current sample tend to have less cooperative behavior (lower SIR and higher RR), which might be independent from social distance. 2) Sample size, the current sample is only as half as the original study, but some of the negative results (e.g. Figure 2C, Figure 3) seems showing similar patterns as the original findings. Besides, the current study also managed to reproduce most of the behavioral findings. Regarding the replication procedure, I am wondering if the author did a power analysis based on Tang et al. (2016). The author might want to specify how they decided the sample size and what they expected if they doubled the sample size.
RESPONSE: I would like to express my sincere appreciation for your valuable comments on our study. Our sample was comprised of individuals who were acquaintances and colleagues from the same laboratory at the time of the study. Therefore, we could not control for the sample size and sampling bias (social distance), as you have mentioned. One of the important future tasks is to conduct a power analysis based on Tang et al. (2016), estimate the required sample size, and clarify the impact of these factors on the results. We have added these points to the limitation sections (Lines 537-543).

3. The authors mentioned two possible reasons for the higher RR as compared to the original study. “However, we could not determine whether the Responder's motivation to reject the offer was altruistic (i.e., to correct the inequitable behavior) or selfish (i.e., to increase their reward).” In my humble opinion, a lower SIR and higher RR seems more likely driven by demands of fairness. If the participant just wanted to maximize the reward, they could accept all the deal no matter to what extent they trust or willing to cooperate with each other, then it should lead to a low RR and low SIR. Moreover, a low RR and low RR does not necessarily link with selfishness. It could be driven by an altruistic motivate as well. For instance, for being pilot, we sometimes might still accept unfair offer, even though we are not willing to. Maybe the authors could use a more specified term for “to correct the inequitable behavior”, instead of the broad term “altruistic”.
RESPONSE: We appreciate and agree with your insightful comment. As you pointed out, the low SIR and high RR may be explained by the demand for fairness. In addition, as shown in Fig. 2D, Proposers had higher gains than Responders regardless of the condition. This suggests that Proposers pursued their own interests, while Responders may have rejected offers and corrected the inequitable behavior. This could explain the low SIR. Therefore, as you pointed out, we have revised the description from the broad term "altruistic" to "to correct the inequitable behavior" (Lines 414-416).

4. It is always encouraged to preregister especially for replication studies. If the authors follow that procedure, it would be good to mention it in the manuscript. If not, it would be good to consider such approach for future studies.
RESPONSE: Thank you for your comment. Indeed, pre-registration is an important scientific method that can enhance reproducibility and reliability. However, we did not conduct pre-registration in this study. In future studies, we will consider the importance of pre-registration and incorporate this method. We have explicitly stated in the manuscript that pre-registration was not conducted in this study (Lines 543-546).

5. I understand that the authors were following the analytical protocol of the original study. I have a concern about the ANOVA analyses, as the measures may be to some extend covaried. For instance, lower fairness rate may associate with higher rejection rate. Therefore, if I were conducting the study, I may consider using MANCOVA instead of ANOVA.
RESPONSE: Thank you for your comment. As you pointed out, we did use ANOVA as the analysis method following the original study. However, we do acknowledge the possibility that the measured variables may have some degree of covariance, as you mentioned. Nevertheless, for the current paper, we believe it is essential to report the results using the same analysis method as the original study. We have added a note in the limitations and future directions section regarding reporting results using MANCOVA to address the covariance issue (Lines 548-553).

6. The current study reported a lower SIR than that of the original study. I am wondering if the author also replicated the finding of the reaction time of during stage 4 (Figure 2B of Tang et al., 2016). The reaction time might provide more insight of certainty of the participants’ judgment.
RESPONSE: We would like to thank the reviewer for this insightful comment. As suggested, we conducted further analysis on the differences in reaction time (RT, referred to as response time in the original study) between conditions. Our results showed that the order of RT between conditions was reversed compared to the original study. While the original study found that participants responded faster in the FF condition, in our study, participants responded faster in the FB condition (Fig. 2E). In the original study, the FF condition involved more verbal communication and facial expressions, which may have contributed to faster RT. On the other hand, our results suggest that in the lower shared intentionality situation, as in our study, it might require more time to infer each other's intention in the FF condition. We have added these discussions to the revised manuscript (Lines 284-286, and 384-399).

7. In the introduction, the author briefly touched Gvirts & Perlmutter (2020). Perhaps it would be helpful if the author could provide simple examples of the three factors mentioned in Gvirts & Perlmutter (2020) for readers who are not familiar with that paper.
RESPONSE: We appreciate the comment on this. We have added simple examples for the three factors, as you recommended (Lines: 59-64).

8. In the last paragraph of introduction, the authors mentioned, “One of the pitfalls of hyperscanning is that …”. Yes, this is a problem recognized in this field and discussed in the literature. Would it be more appropriate to add some reference to this point?
RESPONSE: We appreciate pointing this out. We have cited the following paper for this:
Léné P, Karran AJ, Labonté-Lemoyne E, Sénécal S, Fredette M, Johnson KJ, Léger PM. Is there collaboration specific neurophysiological activation during collaborative task activity? An analysis of brain responses using electroencephalography and hyperscanning. Brain Behav. 2021 Nov;11(11):e2270.

9. How is the measure “gain” defined?
RESPONSE: We appreciate pointing this out. We defined the game gain as the total amount of rewards received normalized by the scores based on the maximum possible reward of ¥47700. We have added the description to Lines 160-162.

10. In the discussion, the authors noted, “we expected the IBS to be greater than in previous studies because we decreased the social distance between the pairs.”. This hypothesis was not mentioned in the introduction.
RESPONSE: We appreciate the feedback and apologize for any confusion. We clarify that the assumption was not a predefined hypothesis but rather an observation made during data analysis. We did not set out to test this assumption specifically but discovered it through exploratory analysis. We have added the following description to Lines 428-432:
Here it is essential to clarify that this assumption is not a predefined hypothesis but rather on exploratory observation conducted after the commencement of the experiment.

11. “Gvirts et al. (2020)” is a two-author paper and should be cited as “Gvirts & Perlmutter (2020)”.
RESPONSE: Thank you for pointing this out. We modified the description as suggested.

[To Reviewer 2]
1. In the manuscript, there is no description of any pre-processing technique for the fNIRS data. Pre-processing is a fundamental part of data analysis and the authors should describe accurately which pre-processing steps were taken (see Yücel et al. 2021. Best practices for fNIRS publications, Neurophotonics). Was the data corrected for motion? Was the data visually inspected for bad channels and for physiological artifacts? If not, how did the authors account for these potential noise sources? Similarly, no information is provided about the software used for statistical analyses (i.e. ANOVAs, t-tests and Friedman test). Could the authors add this information?
RESPONSE: We sincerely appreciate your comments regarding our data preprocessing. We also recognize the importance of Yücel et al. (2021). In our study, we calculated the Wavelet transform coherence (WTC) using the A cross wavelet and wavelet coherence toolbox for MATLAB in the data preprocessing. As in the original study, we assumed we could extract the component due to neural activity using a frequency band of 0.02-0.08 Hz. This has also been stated in the influential fNIRS-hyperscanning study by Cui et al. 2012. Although we did not directly regress out the artifact-related signals in this study, it is expected that the above procedure has contributed to reducing their effect (added in Lines 240-242).

However, we cannot justify having completely removed the presence of potential noise. We agree with the reviewer's opinion on this matter and would like to address it further in future work. To overcome this issue, it is necessary to use devices that directly measure the motion artifact and other confounds (e.g., short-distance channels and accelerometers) to investigate their impact. We have added this description to the revised manuscript in the limitations (added in Lines 563-570).

Additionally, we confirmed the sensor sensitivity with the calibration function of the NIRS device before measuring and excluded the effect of bad channels (added in Lines 182-184). Regarding statistical analyses, such as ANOVA and t-tests, we used MATLAB for them and added this information to Lines 274-276.

2. I In the original study by Tang et al. (2016), the fNIRS analysis was conducted on two specific regions: right TPJ as region of interest and right DLPFC as control region. Is there a specific reason for which the authors of the current manuscript conducted the fNIRS analysis including more extended frontal and temporal regions and did not include a control region? If the aim of the study was to replicate the findings of Tang et al. (2016), why were additional regions included?
RESPONSE: We appreciate your feedback. The DLPFC consists of SFG and MFG (as described in the following two papers) and our probe placement covers the brain regions measured in the original study (Table 1). Additionally, we had the fNIRS device that allows for more channels than the one used in original study. Conducting measurements with more channels and an extended range is effective for reducing the influence of individual differences in head and brain size and avoiding false-negative detection. Therefore, we chose to conduct measurements over a wider range. We have added these points to the manuscript for clarification (Lines 193-200).
[1] Chang CC, Yu SC, McQuoid DR, Messer DF, Taylor WD, Singh K, Boyd BD, Krishnan KR, MacFall JR, Steffens DC, Payne ME. Reduction of dorsolateral prefrontal cortex gray matter in late-life depression. Psychiatry Res. 2011 Jul 30;193(1):1-6. doi: 10.1016/j.pscychresns.2011.01.003. Epub 2011 May 18. PMID: 21596532; PMCID: PMC3105213.
[2] Crespo-Facorro B, Kim J, Andreasen NC, Spinks R, O'Leary DS, Bockholt HJ, Harris G, Magnotta VA. Cerebral cortex: a topographic segmentation method using magnetic resonance imaging. Psychiatry Research. 2000;100(2):97–126.

3. The original study by Tang et al. (2016) had a sample size of 202 participants. The current study used a sample size of 48 participants and the reported effect sizes are quite low. My concern is that the differences in the results obtained may depend on the sample size. How did the authors ensure that the current study was not underpowered? Was a power analysis conducted? Alternatively, I would suggest to mention this in the discussion as a limitation and to add the effect sizes of the fNIRS results.
RESPONSE: We appreciate your insightful comment. The same comment was given by the Reviewer 1. We totally agree with the reviewers’ concern about the sample size. Our participants were recruited based on acquaintances who were enrolled in the same laboratory at the same time. Therefore, we could not control the number of participants and could not exclude the influence of sampling bias (social distance) and sample size, as pointed out by the reviewer. A crucial task for future studies is to conduct power analysis based on Tang et al. (2016), estimate the required sample size, and clarify the impact on the results. We have added these points to the limitation sections (Lines 537-543).
Also, thank you for your suggestion about calculating the effect sizes of the fNIRS results. While effect size is an important statistic in many analyses, it is not a standard output of permutation tests. In the current study, we used permutation tests to compare the coherence changes between paired and pseudo-paired participants. The primary purpose of this analysis was to assess whether the IBS during the task was due to social interaction rather than task-derived effects and to control for potential confounds. While permutation tests can provide a p-value indicating the probability of observing a particular outcome by chance, they do not typically produce effect size estimates. Therefore, calculating effect sizes in the context of permutation tests is not a common practice and may not be meaningful in the current study.

4. The structure of the discussion section (4.1) is somewhat confusing to me. Results, which were significantly tested, and conclusions coming from visual inspections are intermixed throughout the section. In my opinion, in this way it is not clear to the reader, what was statistically evaluated and what was not. I would suggest to separate these observations in different paragraphs: visual inspection of the results, replicated significant and non-significant results and non-replicated results. With regard to the visual inspection of the results (i.e. results which were not statistically evaluated), I refer specifically to the comparison between the current and the original study. No statistically test was used to evaluate the differences between studies, but strong conclusions are drawn (see lines 55-58 of page 24 and lines 43-46 of page 25). I think it is very relevant to compare the results from the previous and the current experiment, but caution should be taken when reporting the observed similarities and differences. These can be speculations and possible interpretations by the authors, but no strong conclusions should be drawn. Alternatively, the authors could statistically test the differences in the results obtained in the original study and in the current manuscript.
RESPONSE: We appreciate your valuable feedback and apologize for the unclear structure of the discussion in Section 4.1. We have followed your suggestion to divide the paragraph and clarify the discussion. Regarding the visual observations, we were unable to statistically compare our results with those of the original study as raw data was not publicly available. Therefore, we designed and analyzed our results independently using the same experimental design and analysis methods as the original study to confirm whether the trends were similar. Nonetheless, as you pointed out, it is not appropriate to make strong conclusions based on this result alone, so we have toned down our claims accordingly. Accordingly, modifications have been made throughout Section 4.1.

5. At the beginning of the discussion on behavioral results, it is reported that the Proposer tended to distribute fairly rewards and the Responder tended to reject the offer (lines 34-40, page 24), with a reference to figure 2A. The figure shows the fairness rate to be between 50% and 60% and the rejection rate between 30% and 40%. Are these considered high values in the context of the ultimatum game? Are these values similar to the ones obtained in the original study?
RESPONSE: Thank you for your insightful comment. We observed a lower fairness rate and higher rejection rate compared to the original study. Initially, we assumed a tendency for fair distribution since the proportion of trials where the reward was distributed fairly exceeded 50%. However, given that the Proposer obtained significantly higher rewards than the Responder, it is likely that fair distribution did not occur, which resulted in a lower SIR and a higher rejection rate. We have added this information to Lines 335-343 for clarification.

6. In the results, the authors report that gain percentage between Proposer and Responder was significant in both conditions (FF and FB) and this is illustrated in a nice graph (figure 2D). Surprisingly, this finding was not mentioned in the discussion. Is there a specific reason for this? I think discussing this finding is extremely relevant and could give further insights on the authors’ hypotheses and conclusions.
RESPONSE: The corresponding part to the following sentence "However, there was a significant difference in gains between the Proposer and Responder in both conditions (FF: p = 0.0012, d = 0.99; FB: p = 0.0003, d = 1.65)" is shown in Fig. 2D. This result suggests that the Proposer tended to pursue their own interests while the Responder tended to reject offers when they believed that the Proposer would behave in this way and demanded fairness. This may also explain the low SIR. These findings have been added to Lines 413-417.

7. In the manuscript, the face-to-face condition is described as a condition in which participants can use non-verbal behavior (eye-gaze and hand gestures). Considering that the availability of non-verbal visual cues is mentioned in the interpretation of the results, I am concerned about the actual visibility of participants’ visual communicative signals. The experiment set-up is nicely illustrated in figure 1A (left). Considering the monitors in front of the participants, I am wondering if participants could actually have a good visibility of their co-participants. I am particularly concerned that hand gestures would not be visible as the gesture space is covered by the monitor and it is hard to judge the visibility of the co-participant’s face from the figure. How did the authors ensure the visibility of the co-participants’ face and that this was similar across pairs? Did the authors record additional data (e.g. eye gaze, video recordings) which could be used to evaluate if the participants were indeed attending to their co-participant’s visual communicative signals?
RESPONSE: We appreciate your insightful feedback. We acknowledge the importance of your point that we still need to provide evidence of whether participants actually utilized visual cues. During the experimental setup, we ensured that participants' facial expressions were visible. We instructed those in the FF condition that they could use the partner's expression as a clue to answer the questions in stage 4. However, we instructed participants to minimize any bodily movements except for pressing the keyboard to avoid the influence of the motion artifact on the results, except for physiological factors (added in Lines 164-169). The visual cues we allowed were limited to the changes in facial expression and the minor movements that could not be controlled. Unfortunately, we could not collect behavioral data to confirm whether visual signals were effectively shared among participants in this study. In future research, it will be important to verify whether information sharing between participants was conducted appropriately by utilizing methods such as eye tracking or video recording. We have added this limitation to Lines 572-578. Also, we have removed the description of ‘gesture’ for clarification.

8. In figure 2C, the y-axis reports the ‘gain percentage’, while the plot likely shows the total amount of money earned by the Proposer and the Responder (as described in line 10-12 on page 27). Could the authors correct the y-axis label?
RESPONSE: We are sorry for the confusing description. In our study, the game gain, the total amount of rewards was calculated as a percentage (%) of the maximum possible reward of ¥47700 (we added its definition in Lines 160-162). The plots (Fig. 2C/D) were correct, but we have updated their caption to be consistent with the words used in the main text.

9. In figure 2D, the gain percentage is significantly different between Proposer and Responder in both conditions, but there are no asterisks indicating the significant relationships. However, asterisks are used to illustrate significant differences in figure 2A. I think the figure would be clearer if significant differences were indicated in the same way throughout the figure.
RESPONSE: We sincerely apologize for the confusing expression. Following your suggestion, we have added asterisks to the necessary parts.

10. In the results section on behavioral data (2.4.1, page 17, lines 37-39), it seems that the shared intentionality rate (SIR) and the positive and negative affect schedule (PNAS) are not considered as behavioral variables. Could the authors clarify this in the manuscript, to avoid causing confusion to the reader?
RESPONSE: We apologize for causing confusion. Following your suggestion, we have added the description of the behavioral measures as follows.
Lines 216-219: The six variables, HR, OP, FR, SIR, RR, Response Time (RT) were analyzed as behavioral metrics. The five behavioral data (HR, OP, FR, RR, RT) were homogeneous in variance (Bartlett's test) and followed a normal distribution (Shapiro–Wilk test).
Lines 227-228: Moreover, the difference in the PANAS score of each pair between the FF and FB conditions was also t-tested.

**Revised Submission - OXFNSC-2022-017.R1**

**Reviewer 1**

Comments to the Author

Review "Interpersonal brain synchronization during face-to-face economic exchange between acquainted dyads" (revision) for Oxford Open Neuroscience.

I can see that the authors have put their effort in addressing my concerns. I especially appreciated that the authors have added a section of limitations and future directions in the revision. However, I still have some comments to add, and hope it could contribute the analysis and layout of the manuscript.

Major comments:

1. The current IBS analysis includes the epochs of stage 2-4. The IBS results were not warrant due to the incompatibility of the FF and FB conditions in stage 2; namely, as the participants only talk in stage 2 in FF condition. It might be worth to do the IBS analysis with the epochs of stage 3-4 (or even stage 3-6), as in those stages the participants were roughly doing the same things in the task.

2. I understand the current study aims at extending the main findings of Tang et al., 2016 to acquaint pairs. However, without directly comparing the original and current studies, or acquiring data from strangers and acquaintances, the current study will not be able to draw a conclusion, though it was a good exploration. The authors might want to discuss it in the limitation.

3. I think the current study is in an exploration stage, so I’d highly suggest the authors to still softer their tone throughout the manuscript (although they already did in the revision), when elicit their hypotheses and interpretation of the original study. Don’t worry it would devalue the quality of your study. Rome city is not built in one day, neither is science. Exploratory study is totally fine. The community will still learn from your experience.

4. For instance, due to the incompatible conditions, I’m afraid a firm conclusion could be drawn.

“Their work is significant, as they demonstrated IBS between two people engaged in FF interactions.”

“FF interaction enhances the shared intentionality between pairs and promotes cooperation because it provides greater visual information about the partner than FB interaction.”

“Therefore, our results suggested that the FF condition or acquaintance with the partner did not affect the Proposer’s behavior regarding fairly distributing the reward.”

“This study had two goals: (1) to replicate the results of Tang et al. (2016) in acquainted pairs, and (2) to demonstrate that IBS during the task is induced by social interaction.”

Minor comments:

5. I also found other somewhat strong statement in this paper, such as “The application of MANCOVA instead of ANOVA is one of the most effective approaches …”. It’s encouraged to make strong statement when it is necessary of course. At the same time, it’s better to keep in mind that, as scientists, we are facing many uncertainties and unknowns most of the time during our career.

6. The interpretation of the reversed RT of the judgement of shared intentionality is not clear to me. The author stated: “in the lower SIR case, as in our more time to infer each in the FF condition”. To my understanding, this statement implied that longer RT in FF condition was a result of lower SIR. However, in the current study, the SIR in FF condition was significantly higher than that of FB condition.

7. Please add the sample size statement also in the section 2.1 Participants.

8. Participants can only communicate verbally in FF condition, not the FB condition, but the description in Line 142-144 sounds like they did in both conditions.

9. The measure ‘game gain’ was missing from Line 216.

10. It might help the reader to capture the main idea of the study quickly, if the authors add a brief summary at the beginning of the 4. Discussion, before digging into the details results.

11. Line 349: I’d suggest “In the FF condition, participants could commutate verbally and nonverbally…”

12. Line 541: remove “, as you have mentioned”

**Reviewer 2**

Comment to the Author

I would like to thank the authors for addressing my concerns and responding to them in a clear and elaborate way. I am confident that the proposed revisions have already improved the manuscript, by making it stronger and more transparent.

As previously mentioned, I think the study presents interesting results providing further insights on the relationship between inter-brain synchronization and social interaction.

Nonetheless, I still have one major concern regarding the pre-processing of the fNIRS data, which I will elaborate below, together with a few minor details, which would further improve the clarity of the manuscript.

1) I appreciate the authors’ response to my concern regarding the unmentioned pre-processing of the fNIRS data: the authors explained that no pre-processing steps were performed, based on the previous influential study by Cui et al. 2012, and the sensor sensitivity was checked before the experiment, to exclude the presence of bad channels.

I thank the authors for clarifying this point, for explaining their reasoning and for adding this to the limitations section of the manuscript. Nonetheless, I still think that pre-processing is a fundamental step of fNIRS data analysis (as explained by Yücel et al., 2021. Best practices for fNIRS publications, Neurophotonics), in particular in light of a replication study.

Considering the authors’ aim of replicating the findings of Tang et al. (2016), it is fundamental to reproduce the experimental and analysis procedure of the original study. Notably, the authors accomplished this in an excellent way with regards to the experiment design (i.e. FF and FB conditions with verbal communication and button presses) and the data analysis (i.e. ANOVA and WTC), but not really with regards to the pre-processing part.

More specifically, Tang et al. (2016) reported the following pre-processing steps on page 26:

after preprocessed with initial time baseline correction, hrf low-pass filtering and wavelet minimum description length detrending in NIRS-SPM (Jang et al., 2009; Ye et al., 2009; Tak et al., 2011), time series of oxy-Hb concentration changes of each channel of each subject were normalized into z-scores with means and standardizations.

In order to claim a replication of the original findings, the authors should follow the same pre-processing procedure, otherwise any difference in the results of the two studies may be due to the different pre-processing approach.

Minor details:

1) The authors outlined the strengths and the limitations of the study in a nicely manner. In the conclusion paragraph, I was confused by the lines 581-583:

“Taking these constraints into consideration, the current study still provides novel insights into the impact of manipulating social distance on IBS, suggesting that higher levels of economic exchange may be associated with greater IBS and that this effect may be modulated by the relationship with the interacting partner.”

As far as the authors report, there were no effect of IBS in relation to the task conditions or the cooperative behavior, but only in relation to the pseudo-pairs analysis. Considering this, it is not clear to me to what finding(s) does the line “higher levels of economic exchange may be associated with greater IBS” refers to. Could the authors clarify this? What do they mean by higher levels of economic exchange? Alternatively, they could modify the sentence to focus on their main findings.

2) I thank the authors for addressing my concern regarding the clarification of the “nonverbal behavior” term mentioned throughout the manuscript. As the authors explained, with “nonverbal behavior” they refer to facial expressions and eye gaze, since the participants were instructed to avoid any other movement. The authors clarified this in every mention of “nonverbal behavior”, besides for the very first time it is introduced (line 349). I would suggest to either define it in its first appearance (as including facial expressions eye gaze) and then simply refer to it as “nonverbal behavior” or alternatively to always refer to it as including facial expressions and eye gaze.

3) The authors reorganized the discussion section in a clearer and organized way, separating observations based on visual inspection and results obtained with statistical testing. However, I feel that the first paragraph of the discussion section may still be a bit too complicated (in particular lines 337-341). I would suggest the authors to rephrase these lines in a simpler way or to divide them in shorter sentences, to ensure that the readers can follow and understand what is being described.

4) In lines 142 and 143, the authors describe the experiment procedure by including that “the proposer was instructed to communicate the total amount verbally”. However, if I understood correctly, this is only true for the FF condition. Therefore, it remains unclear how this information was communicated in the FB condition (via button press?).

To avoid misunderstandings, I would clarify this in the manuscript, for example: “It should be noted that in stages 2 and 3, the Proposer was also instructed to verbally communicate (FF condition only) the total amount (in stage 2) and the transfer amount (in stage 3) besides pressing the button as the response.

5) Lastly, I would like to point out a small typo in line 541: “Therefore, we could not control for the sample size and sampling bias (social distance), as you have mentioned.” I assume the “as you have mentioned” was not supposed to be part of the sentence in the manuscript.

**Decision letter - OXFNSC-2022-017.R1**

25-May-2023

Dear Dr. Hiwa,

Thank for your submitting your revised manuscript OXFNSC-2022-017.R1 entitled "Interpersonal brain synchronization during face-to-face economic exchange between acquainted dyads" to Oxford Open Neuroscience. Your manuscript has been re-reviewed by the original two reviewers.

Both reviewers highlight the improvement to the manuscript, however they also note additional points to consider. I would like you to prepare a further revision in light of these comments.

There are three issues that I think it is essential for you to address in order for your manuscript to be accepted.

First, please tone down any conclusions that go beyond the evidence presented. Both reviewers make helpful suggestions here that I would like you to consider.

Second, there are some methodological details that should be clarified, as noted by reviewer 2.

Third, there are some typos to correct. Reviewer 2 notes the phrase "as you have mentioned" on line 541. I also noticed a couple of issues:

"It is important to note that the game gain, The total amount of rewards" (lines 160-161). Should the first part of this be deleted, or be rephrased in some way?

Table 2: you note several p values equal to zero. It is better to report these as (e.g.) p < .001 rather than as exactly zero.

In addition to these issues, the reviewers make several other helpful points. I would like you to carefully consider these points, but I will not make them conditions for acceptance. In particular, both reviewers suggest some additional analyses (reviewer 1 in terms of the different epochs and reviewer 2 in terms of the pre-processing). I will leave it to you whether you wish to conduct these analyses, however if you do not you should amend the text accordingly, by directly commenting on the issues raised by the reviewers. For example, when discussing the the failure to replicate Tang et al. (2016) you should acknowledge the lack of pre-processing in your study as a potential factor.

Please note that this journal operates with transparent peer review. This means that if your submission is accepted for publication, the full peer review history of your article will publish online alongside your article. This includes reviewer comments, editor decision letters, and your author responses.

To revise your manuscript, log into https://mc.manuscriptcentral.com/oxfnsc and enter your Author Centre, where you will find your manuscript title listed under "Manuscripts with Decisions." Under "Actions," click on "Create a Revision." Your manuscript number has been appended to denote a revision.

You may also click the below link to start the revision process (or continue the process if you have already started your revision) for your manuscript. If you use the below link you will not be required to login to ScholarOne Manuscripts.

*** PLEASE NOTE: This is a two-step process. After clicking on the link, you will be directed to a webpage to confirm. ***

https://mc.manuscriptcentral.com/oxfnsc?URL_MASK=ae5d2668bffd439d8b308e2d41ec0186

You will be unable to make your revisions on the originally submitted version of the manuscript. Instead, revise your manuscript using a word processing program and save it on your computer. Please also highlight the changes to your manuscript within the document by using the track changes mode in MS Word or by using bold or colored text.

Once the revised manuscript is prepared, you can upload it and submit it through your Author Centre.

When submitting your revised manuscript, you will be able to respond to the comments made by the reviewer(s) in the space provided. You can use this space to document any changes you make to the original manuscript. In order to expedite the processing of the revised manuscript, please be as specific as possible in your response to the reviewer(s).

IMPORTANT: Your original files are available to you when you upload your revised manuscript. Please delete any redundant files before completing the submission.

Because we are trying to facilitate timely publication of manuscripts submitted to the Oxford Open Neuroscience, your revised manuscript should be uploaded as soon as possible. If it is not possible for you to submit your revision in a reasonable amount of time, we may have to consider your paper as a new submission.

Once again, thank you for submitting your manuscript to the Oxford Open Neuroscience and I look forward to receiving your revision.

Sincerely,

Professor Sam Gilbert

Senior Editor, Oxford Open Neuroscience

sam.gilbert@ucl.ac.uk

Reviewer(s)' Comments to Author:

Reviewer: 1

Comments to the Author

Review "Interpersonal brain synchronization during face-to-face economic exchange between acquainted dyads" (revision) for Oxford Open Neuroscience.

I can see that the authors have put their effort in addressing my concerns. I especially appreciated that the authors have added a section of limitations and future directions in the revision. However, I still have some comments to add, and hope it could contribute the analysis and layout of the manuscript.

Major comments:

1. The current IBS analysis includes the epochs of stage 2-4. The IBS results were not warrant due to the incompatibility of the FF and FB conditions in stage 2; namely, as the participants only talk in stage 2 in FF condition. It might be worth to do the IBS analysis with the epochs of stage 3-4 (or even stage 3-6), as in those stages the participants were roughly doing the same things in the task.

2. I understand the current study aims at extending the main findings of Tang et al., 2016 to acquaint pairs. However, without directly comparing the original and current studies, or acquiring data from strangers and acquaintances, the current study will not be able to draw a conclusion, though it was a good exploration. The authors might want to discuss it in the limitation.

3. I think the current study is in an exploration stage, so I’d highly suggest the authors to still softer their tone throughout the manuscript (although they already did in the revision), when elicit their hypotheses and interpretation of the original study. Don’t worry it would devalue the quality of your study. Rome city is not built in one day, neither is science. Exploratory study is totally fine. The community will still learn from your experience.

4. For instance, due to the incompatible conditions, I’m afraid a firm conclusion could be drawn.

“Their work is significant, as they demonstrated IBS between two people engaged in FF interactions.”

“FF interaction enhances the shared intentionality between pairs and promotes cooperation because it provides greater visual information about the partner than FB interaction.”

“Therefore, our results suggested that the FF condition or acquaintance with the partner did not affect the Proposer’s behavior regarding fairly distributing the reward.”

“This study had two goals: (1) to replicate the results of Tang et al. (2016) in acquainted pairs, and (2) to demonstrate that IBS during the task is induced by social interaction.”

Minor comments:

5. I also found other somewhat strong statement in this paper, such as “The application of MANCOVA instead of ANOVA is one of the most effective approaches …”. It’s encouraged to make strong statement when it is necessary of course. At the same time, it’s better to keep in mind that, as scientists, we are facing many uncertainties and unknowns most of the time during our career.

6. The interpretation of the reversed RT of the judgement of shared intentionality is not clear to me. The author stated: “in the lower SIR case, as in our more time to infer each in the FF condition”. To my understanding, this statement implied that longer RT in FF condition was a result of lower SIR. However, in the current study, the SIR in FF condition was significantly higher than that of FB condition.

7. Please add the sample size statement also in the section 2.1 Participants.

8. Participants can only communicate verbally in FF condition, not the FB condition, but the description in Line 142-144 sounds like they did in both conditions.

9. The measure ‘game gain’ was missing from Line 216.

10. It might help the reader to capture the main idea of the study quickly, if the authors add a brief summary at the beginning of the 4. Discussion, before digging into the details results.

11. Line 349: I’d suggest “In the FF condition, participants could commutate verbally and nonverbally…”

12. Line 541: remove “, as you have mentioned”

Reviewer: 2

Comments to the Author

I would like to thank the authors for addressing my concerns and responding to them in a clear and elaborate way. I am confident that the proposed revisions have already improved the manuscript, by making it stronger and more transparent.

As previously mentioned, I think the study presents interesting results providing further insights on the relationship between inter-brain synchronization and social interaction.

Nonetheless, I still have one major concern regarding the pre-processing of the fNIRS data, which I will elaborate below, together with a few minor details, which would further improve the clarity of the manuscript.

1) I appreciate the authors’ response to my concern regarding the unmentioned pre-processing of the fNIRS data: the authors explained that no pre-processing steps were performed, based on the previous influential study by Cui et al. 2012, and the sensor sensitivity was checked before the experiment, to exclude the presence of bad channels.

I thank the authors for clarifying this point, for explaining their reasoning and for adding this to the limitations section of the manuscript. Nonetheless, I still think that pre-processing is a fundamental step of fNIRS data analysis (as explained by Yücel et al., 2021. Best practices for fNIRS publications, Neurophotonics), in particular in light of a replication study.

Considering the authors’ aim of replicating the findings of Tang et al. (2016), it is fundamental to reproduce the experimental and analysis procedure of the original study. Notably, the authors accomplished this in an excellent way with regards to the experiment design (i.e. FF and FB conditions with verbal communication and button presses) and the data analysis (i.e. ANOVA and WTC), but not really with regards to the pre-processing part.

More specifically, Tang et al. (2016) reported the following pre-processing steps on page 26:

after preprocessed with initial time baseline correction, hrf low-pass filtering and wavelet minimum description length detrending in NIRS-SPM (Jang et al., 2009; Ye et al., 2009; Tak et al., 2011), time series of oxy-Hb concentration changes of each channel of each subject were normalized into z-scores with means and standardizations.

In order to claim a replication of the original findings, the authors should follow the same pre-processing procedure, otherwise any difference in the results of the two studies may be due to the different pre-processing approach.

Minor details:

1) The authors outlined the strengths and the limitations of the study in a nicely manner. In the conclusion paragraph, I was confused by the lines 581-583:

“Taking these constraints into consideration, the current study still provides novel insights into the impact of manipulating social distance on IBS, suggesting that higher levels of economic exchange may be associated with greater IBS and that this effect may be modulated by the relationship with the interacting partner.”

As far as the authors report, there were no effect of IBS in relation to the task conditions or the cooperative behavior, but only in relation to the pseudo-pairs analysis. Considering this, it is not clear to me to what finding(s) does the line “higher levels of economic exchange may be associated with greater IBS” refers to. Could the authors clarify this? What do they mean by higher levels of economic exchange? Alternatively, they could modify the sentence to focus on their main findings.

2) I thank the authors for addressing my concern regarding the clarification of the “nonverbal behavior” term mentioned throughout the manuscript. As the authors explained, with “nonverbal behavior” they refer to facial expressions and eye gaze, since the participants were instructed to avoid any other movement. The authors clarified this in every mention of “nonverbal behavior”, besides for the very first time it is introduced (line 349). I would suggest to either define it in its first appearance (as including facial expressions eye gaze) and then simply refer to it as “nonverbal behavior” or alternatively to always refer to it as including facial expressions and eye gaze.

3) The authors reorganized the discussion section in a clearer and organized way, separating observations based on visual inspection and results obtained with statistical testing. However, I feel that the first paragraph of the discussion section may still be a bit too complicated (in particular lines 337-341). I would suggest the authors to rephrase these lines in a simpler way or to divide them in shorter sentences, to ensure that the readers can follow and understand what is being described.

4) In lines 142 and 143, the authors describe the experiment procedure by including that “the proposer was instructed to communicate the total amount verbally”. However, if I understood correctly, this is only true for the FF condition. Therefore, it remains unclear how this information was communicated in the FB condition (via button press?).

To avoid misunderstandings, I would clarify this in the manuscript, for example: “It should be noted that in stages 2 and 3, the Proposer was also instructed to verbally communicate (FF condition only) the total amount (in stage 2) and the transfer amount (in stage 3) besides pressing the button as the response.

5) Lastly, I would like to point out a small typo in line 541: “Therefore, we could not control for the sample size and sampling bias (social distance), as you have mentioned.” I assume the “as you have mentioned” was not supposed to be part of the sentence in the manuscript.

**Author response**

We are immensely grateful for the insightful and thoughtful feedback received from the peer reviewers, which greatly improved the quality of our manuscript. We had certain concerns about our study, and the comments from both editors and reviewers helped us identify the limitations of our present work and potential directions for future research. Although our results may not be perfect due to some experimental and analytical concerns, we believe our current findings will significantly contribute to the advancement of social neuroscience.

We have meticulously addressed each of the reviewers' comments in a point-by-point manner. For the convenience of the reviewers, all amendments made in the revised manuscript are highlighted in yellow. We eagerly look forward to your valuable feedback on the revised manuscript.

[To Editor]

1. First, please tone down any conclusions that go beyond the evidence presented. Both reviewers make helpful suggestions here that I would like you to consider.

RESPONSE: We appreciate your guidance. Following your advice and considering the insightful suggestions from both reviewers, we have toned down any conclusions that may have exceeded the evidence presented in our study.

2. Second, there are some methodological details that should be clarified, as noted by reviewer 2.

RESPONSE: For the clarification of methodological details as pointed out by Reviewer 2, please refer to our responses in the section addressed to Reviewer 2. We appreciate your suggestion.

3. Third, there are some typos to correct. Reviewer 2 notes the phrase "as you have mentioned" on line 541. I also noticed a couple of issues: "It is important to note that the game gain, The total amount of rewards" (lines 160-161). Should the first part of this be deleted, or be rephrased in some way?

RESPONSE: We are sorry for any confusion caused. We deleted the phrase “as you have mentioned” and corrected the lines describing the game gain as follows (Lines 162-164):

“It is important to note that the game gain, the total amount of rewards, was calculated as a percentage (%) of the maximum possible reward of ¥47700.”

4. Table 2: you note several p values equal to zero. It is better to report these as (e.g.) p < .001 rather than as exactly zero.

RESPONSE: Thank you for your suggestion. We have modified them as suggested.

5. In addition to these issues, the reviewers make several other helpful points. I would like you to carefully consider these points, but I will not make them conditions for acceptance. In particular, both reviewers suggest some additional analyses (reviewer 1 in terms of the different epochs and reviewer 2 in terms of the pre-processing). I will leave it to you whether you wish to conduct these analyses, however if you do not you should amend the text accordingly, by directly commenting on the issues raised by the reviewers. For example, when discussing the the failure to replicate Tang et al. (2016) you should acknowledge the lack of pre-processing in your study as a potential factor.

RESPONSE: We thank the editor and reviewers for their helpful and constructive comments. We believe it is essential to contribute to the field by presenting the current results and acknowledging their limitations rather than spending time on additional analyses. Of course, the points made by the two reviewers provided insightful and interesting suggestions regarding the analysis. They gave us a clear picture of our current research, its limitations, and future developments. Please refer to the written responses below for detailed responses to each reviewer.

[To Reviewer 1]

1. The current IBS analysis includes the epochs of stage 2-4. The IBS results were not warrant due to the incompatibility of the FF and FB conditions in stage 2; namely, as the participants only talk in stage 2 in FF condition. It might be worth to do the IBS analysis with the epochs of stage 3-4 (or even stage 3-6), as in those stages the participants were roughly doing the same things in the task.

RESPONSE: We are grateful for the insightful comments from the reviewer. Our study analyzed Interpersonal Brain Synchronization (IBS) in stages 2-4, the same analysis epochs as in the original study. The participant (Proposer) verbally conveyed the amount in stages 2 and 3 only in the FF condition. Indeed, this may affect the warranty of the current IBS results due to the incompatibility between FF and FB conditions. Therefore, observing IBS in only those stages with high compatibility between conditions, as the reviewer suggests, is indeed very intriguing. However, rather than performing these analyses and interpretations with our current data, we believe it would be more reliable to design a new experiment with completely identical tasks.

Thus, considering the focus of the current study, which is to observe the difference in the same analysis interval as the original research, we would like to consider the analysis suggested by the reviewer for future work. We have added these considerations to the limitations and future directions (Lines 586-589):

“Also, since only stages 2 and 3 included verbal communication only for the FF condition, it is worth analyzing IBS in other stages except for these epochs for higher compatibility in the task between conditions.”

Again, we would like to express our deep gratitude for providing us with such an intriguing perspective.

2. I understand the current study aims at extending the main findings of Tang et al., 2016 to acquaint pairs. However, without directly comparing the original and current studies, or acquiring data from strangers and acquaintances, the current study will not be able to draw a conclusion, though it was a good exploration. The authors might want to discuss it in the limitation.

RESPONSE: We agree with the reviewer's opinion. Indeed, the data we have measured is insufficient to lead to strong conclusions regarding the aims of our study. Reviewer 2 expressed similar concerns, and we also recognize the need for discussion on the limitations that conclusions drawn from the current study results cannot be directly compared with the original study and on the next steps, such as measuring both the data of strangers and acquaintances. We have added these points to the 'Limitations' section (Lines 608-616):

“Fifth, our current study's design and dataset do not permit a direct comparison with the original study conducted by Tang et al., 2016 due to different probe settings and monetary units in representing the reward. Indeed, for more conclusive insights, it would be crucial to collect and directly compare data from both acquainted pairs and strangers. Such a comparative analysis would bolster the reliability of our current findings and yield a more comprehensive understanding of the disparities in inter-brain synchronization between these two groups. Pursuing this approach presents a worthwhile direction for future research endeavors.”

3. I think the current study is in an exploration stage, so I’d highly suggest the authors to still softer their tone throughout the manuscript (although they already did in the revision), when elicit their hypotheses and interpretation of the original study. Don’t worry it would devalue the quality of your study. Rome city is not built in one day, neither is science. Exploratory study is totally fine. The community will still learn from your experience. For instance, due to the incompatible conditions, I’m afraid a firm conclusion could be drawn.

 “Their work is significant, as they demonstrated IBS between two people engaged in FF interactions.”

 “FF interaction enhances the shared intentionality between pairs and promotes cooperation because it provides greater visual information about the partner than FB interaction.”

 “Therefore, our results suggested that the FF condition or acquaintance with the partner did not affect the Proposer’s behavior regarding fairly distributing the reward.”

 “This study had two goals: (1) to replicate the results of Tang et al. (2016) in acquainted pairs, and (2) to demonstrate that IBS during the task is induced by social interaction.”

RESPONSE: Thanks to the reviewer's thoughtful encouragement and comments, we have re-recognized the positioning of our current research and the issues we should solve for the next step. As you have confirmed, we are aware of the limitations of our current research results and have already toned down our assertions in the first revision. We believe that our current study is still in the exploratory stage and that we can contribute to the community by clarifying what limitations exist in our current results. We have again thoroughly revised the Discussion, making sure to only write as conclusions we can assert after explicitly stating in the 'Limitations' section that this is an exploratory study. Besides, regarding each of the four parts extracted by the reviewer, we have certainly rephrased these statements to soften their assertions:

- "Their work is significant, as they demonstrated IBS between two people engaged in FF interactions."

--> Revised: "Their work provides important insights, as they observed IBS between two individuals during FF interactions."

- "FF interaction enhances the shared intentionality between pairs and promotes cooperation because it provides greater visual information about the partner than FB interaction."

--> Revised: "FF interaction may potentially enhance shared intentionality between pairs and promote cooperation, as it offers more visual information about the partner compared to FB interaction."

- "Therefore, our results suggested that the FF condition or acquaintance with the partner did not affect the Proposer’s behavior regarding fairly distributing the reward."

--> Revised: "Therefore, our results indicate a possibility that the FF condition or acquaintance with the partner might not significantly impact the Proposer’s behavior in terms of fair reward distribution."

- "This study had two goals: (1) to replicate the results of Tang et al. (2016) in acquainted pairs, and (2) to demonstrate that IBS during the task is induced by social interaction."

--> Revised: "This study aimed to (1) explore if the results of Tang et al. (2016) can be observed in acquainted pairs, and (2) investigate whether IBS during the task could be influenced by social interaction."

4. I also found other somewhat strong statement in this paper, such as “The application of MANCOVA instead of ANOVA is one of the most effective approaches …”. It’s encouraged to make strong statement when it is necessary of course. At the same time, it’s better to keep in mind that, as scientists, we are facing many uncertainties and unknowns most of the time during our career.

RESPONSE: We thank the reviewer for the insightful comment. We agree that it's important to acknowledge the inherent uncertainties and unknowns in scientific research. To reflect this, we have revised our statement regarding the use of MANCOVA to make it less definitive, and we have added a discussion on its limitations and potential drawbacks. We appreciate your guidance in improving the balance and accuracy of our manuscript (Lines 573-578):

“Applying MANCOVA instead of ANOVA could potentially be an effective approach to address this issue and should be considered in future research. However, while MANCOVA can control for confounding variables, interpreting its results can be challenging and may not be appropriate if certain assumptions are not met. Considering ways to handle covariates would be a significant issue in enhancing the reliability of the results.”

5. The interpretation of the reversed RT of the judgement of shared intentionality is not clear to me. The author stated: “in the lower SIR case, as in our more time to infer each in the FF condition”. To my understanding, this statement implied that longer RT in FF condition was a result of lower SIR. However, in the current study, the SIR in FF condition was significantly higher than that of FB condition.

RESPONSE: We appreciate the reviewer’s insightful comment. We apologize for the confusion regarding our interpretation of the SIR values. We were not suggesting a comparison in SIR between conditions in our study, but rather, we were comparing our results with those from previous research. In our statement, "in the lower SIR case, as in our study, more time to infer each other in the FF condition,” we intended to highlight that the lower SIR value in our study, as compared to previous studies, may have led to a longer response time in the FF condition. We realize our phrasing may have been ambiguous, and we appreciate your feedback to improve our explanation. We will clarify this in the revised manuscript as follows (Lines 402-411):

“On the other hand, although it is based on visual observation, our result exhibited a tendency for lower SIR and higher RR, which is opposite to those of the original study. These results suggest that pairs could effectively use external visible cues such as facial expressions in situations with higher shared intentionality. In contrast, when SIR is lower, as observed in our study compared to the original study, it might require more time to infer each other's intentions in the FF condition. Importantly, while the SIR value was indeed higher in the FF condition than the FB condition in our study, both were lower than those reported in the original study.”

6. Please add the sample size statement also in the section 2.1 Participants.

RESPONSE: We thank the reviewer for pointing this out. We have added the following description to section 2.1 Participants (Lines 100-104):

“All pairs belonged to the same laboratory at the time of the study and were acquainted before the experiment; they recognized each other's names and faces and had daily conversations. It should be noted that we could not control for the sample size and sampling bias, such as social distance.”

7. Participants can only communicate verbally in FF condition, not the FB condition, but the description in Line 142-144 sounds like they did in both conditions.

RESPONSE: I apologize for any confusion caused. The relevant sentences have been amended to include "in the FF condition only" as follows (Lines 144-146):

“It should be noted that in stages 2 and 3, the Proposer was also instructed to verbally communicate (in the FF condition only) the total amount (in stage 2) and the transfer amount (in stage 3) besides pressing the button as the response.”

8. The measure ‘game gain’ was missing from Line 216.

RESPONSE: As pointed out, we have added 'game gain' as the behavioral measure (Lines 218-219):

“The six variables, HR, OP, FR, SIR, RR, game gain, and Response Time (RT) were analyzed as behavioral metrics.”

We appreciate the feedback from the reviewer.

9. It might help the reader to capture the main idea of the study quickly, if the authors add a brief summary at the beginning of the 4. Discussion, before digging into the details results.

RESPONSE: We appreciate the reviewer’s valuable suggestion. As suggested, we have added a brief summary at the beginning of each subsection in the Discussion.

Lines 337-342: “Here, we examined the role of shared intentionality in face-to-face and face-blocked interactions, with a particular focus on the fairness and acceptance of reward distribution in acquainted pairs. In the following sections, we delve into the details of our results, discussing in sequence the replicated significant results, the replicated non-significant results, and the findings that were not replicated.”

Lines 432-442: “In the following discussion, we delve into our findings related to IBS in FF and FB interaction conditions. We explore why our results differ from the original study, focusing on the influence of social distance on participants' cooperative behavior and IBS. The specific brain regions associated with these interactions and their implications are discussed. Also, our study's strengths are addressed, including its novel exploration of social interaction's effect on IBS and the successful identification of pair-specific IBS regions. In addition, we acknowledge and discuss the limitations we encountered during our research, covering potential sampling bias, methodological challenges, and interpretational issues. Finally, we look ahead to future directions of the current study.”

10. Line 349: I’d suggest “In the FF condition, participants could commutate verbally and nonverbally…”

RESPONSE: We have made the corrections as indicated. We thank the reviewer for the suggestion (Lines 357-359):

“In the FF condition, participants could communicate verbally and nonverbally (e.g., eye contact and facial expression) in real-time, which may have facilitated an increase in the SIR.”

11. Line 541: remove “, as you have mentioned”

RESPONSE: We apologize for the typographical error and any confusion it may have caused. We have removed it (Line 564).

[To Reviewer 2]

1. I appreciate the authors’ response to my concern regarding the unmentioned pre-processing of the fNIRS data: the authors explained that no pre-processing steps were performed, based on the previous influential study by Cui et al. 2012, and the sensor sensitivity was checked before the experiment, to exclude the presence of bad channels. I thank the authors for clarifying this point, for explaining their reasoning and for adding this to the limitations section of the manuscript. Nonetheless, I still think that pre-processing is a fundamental step of fNIRS data analysis (as explained by Yücel et al., 2021. Best practices for fNIRS publications, Neurophotonics), in particular in light of a replication study. Considering the authors’ aim of replicating the findings of Tang et al. (2016), it is fundamental to reproduce the experimental and analysis procedure of the original study. Notably, the authors accomplished this in an excellent way with regards to the experiment design (i.e. FF and FB conditions with verbal communication and button presses) and the data analysis (i.e. ANOVA and WTC), but not really with regards to the pre-processing part. More specifically, Tang et al. (2016) reported the following pre-processing steps on page 26: after preprocessed with initial time baseline correction, hrf low-pass filtering and wavelet minimum description length detrending in NIRS-SPM (Jang et al., 2009; Ye et al., 2009; Tak et al., 2011), time series of oxy-Hb concentration changes of each channel of each subject were normalized into z-scores with means and standardizations. In order to claim a replication of the original findings, the authors should follow the same pre-processing procedure, otherwise any difference in the results of the two studies may be due to the different pre-processing approach.

RESPONSE: We appreciate the reviewer's keen attention to the importance of preprocessing in fNIRS data analysis and the explicit mention of its necessity in the context of replication studies. We concur that our decision not to preprocess the data, diverging from the approach of the original study by Tang et al. (2016), potentially affected our results and partially explains why some of the original study's findings were not replicated.

We would like to clarify that we fully acknowledge the significance of this point, and it was not our intention to overlook or downplay its importance. Rather, our choice was influenced by the precedent set by influential studies like Cui et al., 2012, in which no preprocessing steps were undertaken.

We have acknowledged this limitation in our manuscript and have now added an extended discussion on the absence of preprocessing in our study (Lines 598-606):

“Furthermore, the current study chose not to preprocess the data, a decision that diverged from the approach of the original study by Tang et al. (2016). Our choice was influenced by the precedent set by influential studies like Cui et al., 2012, in which no preprocessing steps were undertaken. However, a recent study (Yücel et al., 2021) states that preprocessing is a fundamental step of fNIRS data analysis. We acknowledge that these factors potentially affected our results and partially explain why some of the original study's findings were not replicated. Replicating the original study at a more detailed level of preprocessing represents important areas for future work.”

We believe that this clarification enhances the transparency of our methodological approach and encourages awareness of the limitations. We thank the reviewer once again for emphasizing this critical point.

2. The authors outlined the strengths and the limitations of the study in a nicely manner. In the conclusion paragraph, I was confused by the lines 581-583: “Taking these constraints into consideration, the current study still provides novel insights into the impact of manipulating social distance on IBS, suggesting that higher levels of economic exchange may be associated with greater IBS and that this effect may be modulated by the relationship with the interacting partner.” As far as the authors report, there were no effect of IBS in relation to the task conditions or the cooperative behavior, but only in relation to the pseudo-pairs analysis. Considering this, it is not clear to me to what finding(s) does the line “higher levels of economic exchange may be associated with greater IBS” refers to. Could the authors clarify this? What do they mean by higher levels of economic exchange? Alternatively, they could modify the sentence to focus on their main findings.

RESPONSE: We appreciate the reviewer's keen observation and agree with the confusion arising from lines 581-583 in the previous version of our manuscript. Indeed, the original phrasing might have given a misleading impression about the scope and results of our study. We have taken your comments to heart and revised the concluding paragraph to reflect our key findings more accurately. The revised paragraph now reads (Lines 626-637):

"Taking these constraints into consideration, our study contributes to the field of social neuroscience by providing new perspectives on the influence of social interactions on inter-brain synchronization (IBS). Contrary to the original study's findings, our results did not demonstrate any task-induced IBS increase or correlation with cooperative behavior. Instead, our random pair analysis revealed significant pair-specific IBS only under the task condition, observable in the left and right SFG, MFG, ORBsup, and the right STG, PreCG, and PoCG. These findings tentatively suggest that face-to-face interaction in acquainted pairs might not increase IBS, hinting at the possibility that the nature of IBS could be influenced by whom we interact with and how. As such, we cautiously propose that the current study might offer some preliminary insights into the complex dynamics of social distance manipulation on IBS."

We hope this revision provides a clearer representation of our work and its contributions. We thank the reviewer once again for their insightful comments, which have greatly enhanced the clarity and accuracy of our manuscript.

3. I thank the authors for addressing my concern regarding the clarification of the “nonverbal behavior” term mentioned throughout the manuscript. As the authors explained, with “nonverbal behavior” they refer to facial expressions and eye gaze, since the participants were instructed to avoid any other movement. The authors clarified this in every mention of “nonverbal behavior”, besides for the very first time it is introduced (line 349). I would suggest to either define it in its first appearance (as including facial expressions eye gaze) and then simply refer to it as “nonverbal behavior” or alternatively to always refer to it as including facial expressions and eye gaze.

RESPONSE: We agree with the reviewer's suggestion. As proposed, we have now defined 'nonverbal behavior' to include facial expressions and eye gaze at its first mention (Line: 358), and subsequently, we simply referred to it as 'nonverbal behavior.' We thank the reviewer for pointing out this necessary clarification.

4. The authors reorganized the discussion section in a clearer and organized way, separating observations based on visual inspection and results obtained with statistical testing. However, I feel that the first paragraph of the discussion section may still be a bit too complicated (in particular lines 337-341). I would suggest the authors to rephrase these lines in a simpler way or to divide them in shorter sentences, to ensure that the readers can follow and understand what is being described.

RESPONSE: We appreciate both reviewers for their valuable feedback on the complexity of the discussion section. We've considered the reviewers' suggestions and added a brief summary at the beginning of each subsection in the discussion. Also, the complicated sentences the reviewer pointed out were rephrased as follows.

Lines 337-342: Here, we examined the role of shared intentionality in face-to-face and face-blocked interactions, with a particular focus on the fairness and acceptance of reward distribution in acquainted pairs. In the following sections, we delve into the details of our results, discussing in sequence the replicated significant results, the replicated non-significant results, and the findings that were not replicated.

Lines 345-349: More than 50% of trials were allocated to the fair condition, which might imply a fair or near-fair distribution. However, Fig. 2D displays significantly higher earnings for the Proposer compared to the Responder, suggesting a fair distribution was not effectively achieved.

Lines 432-442: In the following discussion, we delve into our findings related to IBS in FF and FB interaction conditions. We explore why our results differ from the original study, focusing on the influence of social distance on participants' cooperative behavior and IBS. The specific brain regions associated with these interactions and their implications are discussed. Also, our study's strengths are addressed, including its novel exploration of social interaction's effect on IBS and the successful identification of pair-specific IBS regions. In addition, we acknowledge and discuss the limitations we encountered during our research, covering potential sampling bias, methodological challenges, and interpretational issues. Finally, we look ahead to future directions of the current study.

We believe this modification will enhance the readability and clarity of our manuscript.

5. In lines 142 and 143, the authors describe the experiment procedure by including that “the proposer was instructed to communicate the total amount verbally”. However, if I understood correctly, this is only true for the FF condition. Therefore, it remains unclear how this information was communicated in the FB condition (via button press?). To avoid misunderstandings, I would clarify this in the manuscript, for example: “It should be noted that in stages 2 and 3, the Proposer was also instructed to verbally communicate (FF condition only) the total amount (in stage 2) and the transfer amount (in stage 3) besides pressing the button as the response.

RESPONSE: We have made the corrections as indicated. We thank the reviewer for the suggestion (Lines 144-146):

“It should be noted that in stages 2 and 3, the Proposer was also instructed to verbally communicate (in the FF condition only) the total amount (in stage 2) and the transfer amount (in stage 3) besides pressing the button as the response.”

6. Lastly, I would like to point out a small typo in line 541: “Therefore, we could not control for the sample size and sampling bias (social distance), as you have mentioned.” I assume the “as you have mentioned” was not supposed to be part of the sentence in the manuscript.

RESPONSE: We apologize for the typographical error and any confusion it may have caused. We have removed it (Line 564).

**Revised Submission - OXFNSC-2022-017.R2**

**Decision letter - OXFNSC-2022-017.R2**

15-Jun-2023

Dear Dr. Hiwa,

It is a pleasure to accept your manuscript entitled "Interpersonal brain synchronization during face-to-face economic exchange between acquainted dyads" in its current form for publication in the Oxford Open Neuroscience.

Please note that this journal operates with transparent peer review. This means that the full peer review history of your article will publish online alongside your article. This includes reviewer comments, editor decision letters, and your author responses.

Next steps

You will receive an email from no-reply@scipris.com within roughly one week. This is your invitation to sign up for an account with SciPris, Oxford University Press’ author portal hosted by Aptara. You will need to create an account if you do not already hold one. Please register or log into your account and follow the online instructions which will guide you through signing your licence and paying the APC. The email and the portal have clearly signposted support options if you need any help during this process.

Please note that SciPris is a completely different system from ScholarOne, so your credentials to submit your manuscript here will not work there. Once you’ve created a SciPris account, you will be able to use it whenever you publish with Oxford Open Neuroscience or any OUP journal. Please note that OUP will only ever request payment for applicable fees be made via SciPris or to an OUP bank account. If you ever have concerns about the legitimacy of a request, please do not hesitate to contact a customer services agent via the SciPris portal or directly via oupsupport@scipris.com.

Thank you for your fine contribution. On behalf of the Editors of the Oxford Open Neuroscience, we look forward to your continued contributions to the Journal.

Sincerely,

Professor Sam Gilbert

Senior Editor, Oxford Open Neuroscience

sam.gilbert@ucl.ac.uk

Reviewer(s)' Comments to Author:
